# Multiple Molecular Dynamics Simulations and Energy Analysis Unravel the Dynamic Properties and Binding Mechanism of Mutants HIV-1 Protease with DRV and CA-p2

**DOI:** 10.1128/spectrum.00748-21

**Published:** 2022-03-23

**Authors:** Ruige Wang, Qingchuan Zheng

**Affiliations:** a Institute of Theoretical Chemistry, College of Chemistry, Jilin University, Changchun, People's Republic of China; University of Sussex

**Keywords:** HIV-1 PR, drug resistance, MD simulation, MM-PBSA analyses, solvated interaction energy analyses

## Abstract

PR^S17^, a variant of human immunodeficiency virus type I protease (HIV-1 PR), has 17 mutated residues showing high levels of multidrug resistance. To describe the effects of these mutated residues on the dynamic properties and the binding mechanism of PR with substrate and inhibitor, focused on six systems (two complexes of WT PR and PR^S17^ with inhibitor Darunavir (DRV), two complexes of WT PR and PR^S17^ with substrate analogue CA-p2, two unligand WT PR and PR^S17^), we performed multiple molecular dynamics (MD) simulations combined with MM-PBSA and solvated interaction energy (SIE) methods. For both the unligand PRs and ligand-PR complexes, the results from simulations revealed 17 mutated residues alter the flap-flap distance, the distance from flap regions to catalytic sites, and the curling degree of the flap tips. These mutated residues changed the flexibility of the flap region in PR, and thus affected its binding energy with DRV and CA-p2, resulting in differences in sensitivity. Hydrophobic cavity makes an important contribution to the binding of PR and ligands. And most noticeable of all, the binding of the guanidine group in CA-p2 and Arg8’ of PR^S17^ is useful for increasing their binding ability. These results have important guidance for the further design of drugs against multidrug resistant PR.

**IMPORTANCE** Developing effective anti-HIV inhibitors is the current requirement to cope with the emergence of the resistance of mutants. Compared with the experiments, MD simulations along with energy calculations help reduce the time and cost of designing new inhibitors. Based on our simulation results, we propose two factors that may help design effective inhibitors against HIV-1 PR: (i) importance of hydrophobic cavity, and (ii) introduction of polar groups similar to the guanidine group.

## INTRODUCTION

Human immunodeficiency virus (HIV) destroys the immune system, bringing about AIDS (1–3). At present, highly active antiretroviral therapy (HAART) consisting of several small molecule drugs is currently the most effective treatment method (4, 5). At different stages of its life cycle, the drugs can target and attack the virus, thereby preventing its replication and reducing damage to the immune system (6, 7). However, the emergence of mutant HIV strains renders these drugs ineffective (8, 9). Therefore, there is an increasing need to develop new drugs against AIDS.

**FIG 1:**
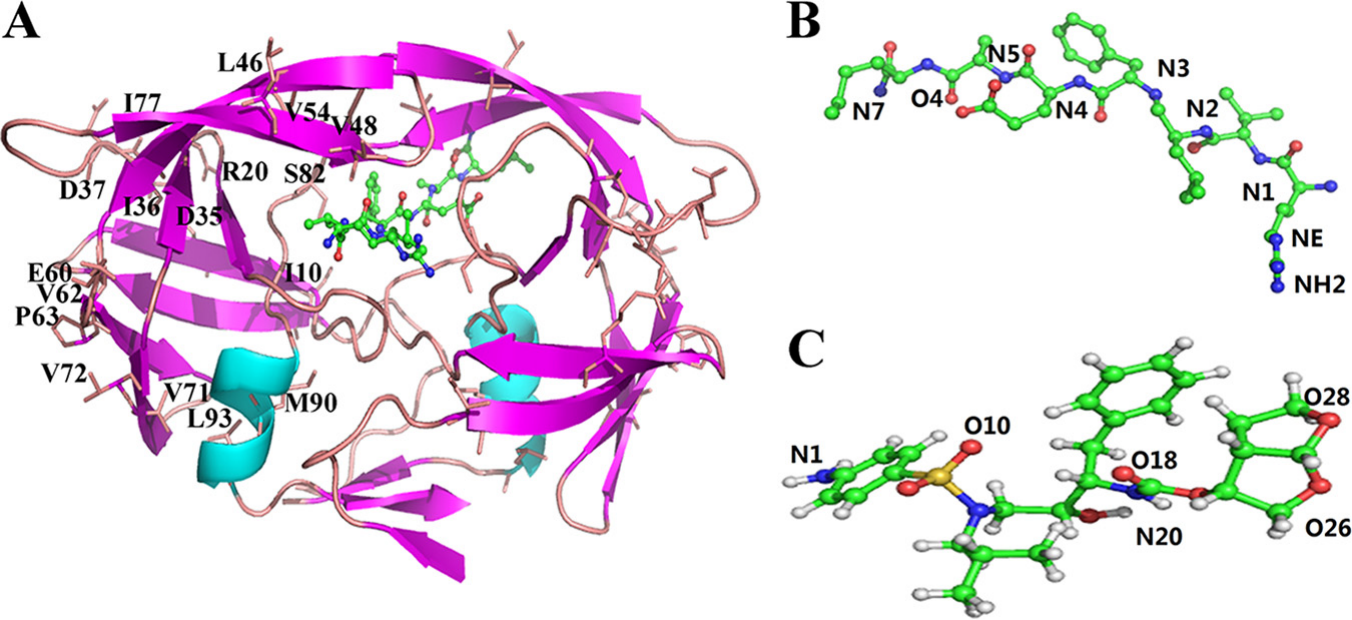
The structure of HIV-1 PR bound to the ligand CA-p2 (PDB ID: 6O48, A) and the ligands (B. CA-p2, C. DRV) used in this study.

The main function of HIV-1 protease (PR) is to cleave Gag and Gag-Pol polyproteins to yield the structural and functional proteins, and then further generate mature infectious HIV particles (10–12). Therefore, PR is an important target in the design of anti-AIDS drugs. Currently, a variety of PR inhibitors has been approved in anti-HIV clinical treatment (13, 14). The development of resistance variants reduces the sensitivity to PR inhibitors and makes the HAART ultimately ineffective (15, 16). Therefore, developing effective anti-HIV inhibitors is the current requirement to cope with the emergence of the resistance (17–19). The work of Weber et al. identified the structure of clinical variant PR^S17^ with 17 mutated residues (Fig. 1A) (20, 21). These mutated residues (L10I, K20R, E35D, M36I, S37D, M46L, G48V, I54V, D60E, I62V, I63P, A71V, I72V, V77I, V82S, L90M, and I93L) make PR extremely resistant to almost all clinical available drugs including DRV. Among these residues, only one residue (V82S) is present in the binding pocket and residues (M46L, G48V, and I54V) are in the flap region. The other residues are located far away from the active site. Interesting, the results of Weber et al. using isothermal titration calorimetry show that PR^S17^ exhibits increased binding to substrate analogue CA-p2 relative to wild type (WT) PR (20). Thus, elucidating the binding mechanism of PR^S17^ with DRV and CA-p2 (Fig. 1B), and understanding the conformational changes caused by these mutated residues can help design new anti-HIV drugs.

Molecular dynamics (MD) simulations along with energy calculations have been widely used to inquiry the critical interaction of ligand-receptor systems (22–28). These methods are not only used to understand the plentiful dynamical structural information of macromolecular complexes, but also to predict their binding affinity (29–31). Compared with the experiments, these methods help reduce the time and cost of designing new drugs (32–33). However, the conformations sampled from a single MD simulation may drop into a locally minimal space, resulting in insufficient conformational sampling (34). Actually, multiple MD simulations can better sample the simulated conformations (34–39). Therefore, in this work, multiple MD simulations and energy analysis were carried out to understand the effects of 17 mutated residues on the structural properties of PR and the interaction of PR-ligand. Firstly, MD simulations were conducted for six systems (two complexes of WT PR and PR^S17^ with DRV, two complexes of WT PR and PR^S17^ with CA-p2, two unligand WT PR and PR^S17^) to gain the dynamic structural information. Secondly, binding free energies were calculated using molecular mechanics Poisson-Boltzmann surface area (MM-PBSA) (40–42), and solvated interaction energy (SIE) approaches (43) to examine the effects of mutated residues on the binding affinity of PR with DRV and CA-p2. Thirdly, the energy decomposition approach was used to study the effects of mutated residues on the detailed interaction and the contribution of individual residues. During this work, we mainly focused on the following aspects: (i) describing the effects of mutated residues on the structure changes of PR, thereby affecting the binding ability of PR with DRV or CA-p2, (ii) predicting the energies of the four PR-ligand complexes and exploring the important residues in the interaction, and (iii) investigating the reason for the difference in the sensitivity of PR to DRV and CA-p2.

## RESULTS AND DISCUSSION

### Stability and flexibility of unligand PR and PR-ligand complexes.

To evaluate the stability of simulations, root mean square deviation (RMSD) of backbone atoms was calculated (Fig. S1 and Fig. S2). On the whole, after about 50 ns simulations, RMSD in each MD trajectory reaches equilibrium, indicating all studied systems are stable. For the unligand PRs, PR^S17^ has a higher average value (2.38 Å) than WT PR (1.69 Å), indicating PR^S17^ is unstable and the structure changes more obviously. These structural changes of unligand PR can be also seen from initial conformations and extract conformations by clustering analysis from simulations (Fig. S3). These structures are similar to the crystal structure of semi-open conformation of unligand WT PR and the open conformation of unligand PR^S17^ (44, 45). When bound to DRV, WT PR was particularly stable with a mean value about 1.24 Å, but for variant PR^S17^, mutated residues promoted the instability of interaction between PR and DRV, which is reflected in the higher mean value about 1.32 Å. This indicates that the mutated residues may have induced structural changes to the complex. However, when bound to CA-p2, WT PR has higher mean values (1.47 Å) than that of PR^S17^ (1.33 Å). The relatively smaller RMSD value of PR^S17^ may be reflected in the strengthening of interaction between PR^S17^ and CA-p2. Moreover, these small mean RMSD values of PR indicated that these four complexes have no significant changes. To further confirm the stability of simulated trajectories, time evolution of enthalpy and entropy were plotted for the equilibrated trajectories of three replicas (600 ns). As shown in Fig. S4 and S5, the calculated enthalpies and entropies exhibit slightly large fluctuations among MD snapshots, but their accumulated mean values quickly became stable in all four complexes. Therefore, the equilibrated 600 ns trajectories can be performed for post process analysis.

To compare the differences in PR flexibility, root mean square fluctuations (RMSF) of C*α* atom was calculated (Fig. S6). Generally, the flexibility pattern of residues in PR^S17^ is similar to that of WT PR. The major differences of residues occur in the fulcrum (around 16 and 16’), flap elbow (around 41 and 41’), and flap region (49 to 52 and 49’ to 52’). These regions in unligand PRs (Fig. S6A) show high flexibility, but their RMSF are decreased when binding with ligands (Fig. S6B and S6C). This result may be caused by the interaction between PR and ligands, which makes the rigidity of PR in the complexes. For DRV-PR complexes, the higher RMSF of PR^S17^ is related to the relatively large conformational fluctuations (Fig. S6B). However, for CA-p2-PR complexes, the lower flexibility of PR^S17^ implies that PR^S17^ may have the higher binding affinity with CA-p2. In short, the difference in PR flexibility of the above-mentioned residues may affect the binding between PR and DRV or CA-p2.

As shown in Fig. S7, compared with unligand WT PR, 17 mutated residues caused a reduction in the correlated movement of R1 and R3 in PR^S17^, and enhanced the correlated movement of its region R2. Relative to DRV-WT PR, these mutated residues in PR^S17^ not only reduced the correlated movement of R1 and R3, but also enhanced the correlated movement of R2. Relative to residues 60 to 80, mutated residues also weaken the anticorrelated movement of residues 80 to 120. Compared with CA-p2-WT PR, these mutated residues in PR^S17^ not only caused a significant reduction in the correlated movement of R1 and R3, but also enhanced the correlated movement of R2. Briefly, these 17 mutated residues promote changes in the conformation of PR flaps, consequently affecting the binding between PR and DRV or CA-p2.

As can be seen from Fig. S8, relative to unligand WT PR, angles *φ* and *Ψ* of residue 50 in chain A of PR^S17^ have changed significantly, but angle *φ* of residue 50 in chain B has changed about 10°. For DRV-PR^S17^, relative to DRV-WT PR, angles *φ* and *Ψ* of residue 50 in chain A change about 50°. These mutated residues do not cause a significant change in angle *φ* of residue 50 in chain B, but they cause a change of about 40° in angle *Ψ* of residue 50. For CA-p2-PR^S17^, relative to CA-p2-WT PR, angle *Ψ* of residues 50 in chains A and B has changed about 20°, while angle *φ* of residue 50 has a significant change. Altogether, 17 mutated residues caused certain changes in angles *φ* and *Ψ* of residue 50, which affected the conformational changes of the PR flaps and the binding between PR and DRV or CA-p2.

### Local fluctuation for unligand PR and PR-ligand complexes.

**FIG 2:**
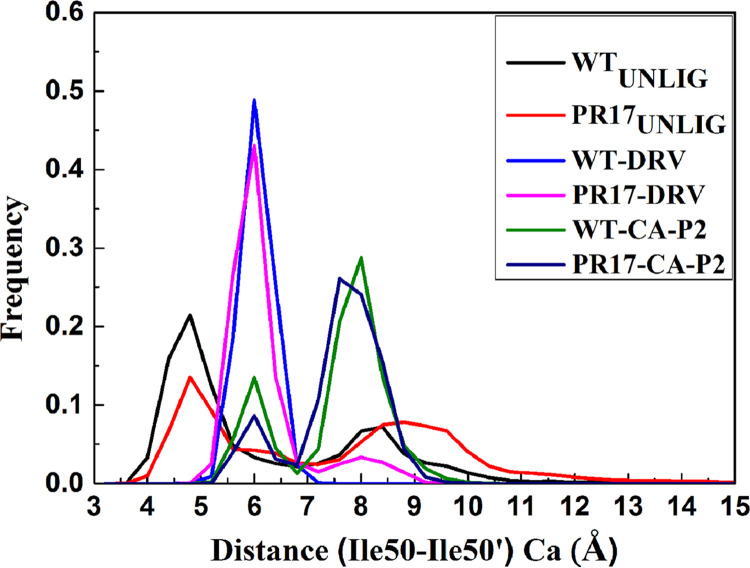
Histogram distributions of Ile50-Ile50’ distance in unligand PR and ligand bound PR complexes.

Distribution of distance between Cα atoms of Ile50 and Ile50’ was drawn to explore the motion of flap tips (Fig. 2). The unligand WT and mutant PR adopt two different sampling structures, the main peak is near 4.80 Å and the other peak is near 8.5 Å. The mean value (standard deviation) of WT PR is 6.29 Å (2.02 Å), and that of PR^S17^ is 7.49 Å (2.44 Å). For DRV-WT/mutant PR, the peaks locate near 6.0 Å. The mean value (standard deviation) of WT PR is 6.01 Å (0.76 Å), that of PR^S17^ is 6.48 Å (0.85 Å), respectively. For CA-p2-WT/mutant PR, the main peak is near 8 Å, and the other minor is near 6.0 Å. The mean value (standard deviation) of WT PR is 7.20 Å (1.52 Å), that of PR^S17^ is 6.93 Å (0.72 Å), respectively. Clearly, the fluctuation of the distance between flap tips in PR-ligand complexes is smaller than that in unligand PR. From the structures obtained by cluster analysis (Fig. S9), we can also see the above-mentioned different mobility of the flaps in WT PR and PR^S17^. Apparently, the distance of Ile50-Ile50’ in PR^S17^-CA-p2 complex fluctuates less than that of other PRs. Thus, when binding with CA-p2, the active site is less open in PR^S17^, and the binding between CA-p2 and PR^S17^ is tighter.

**FIG 3:**
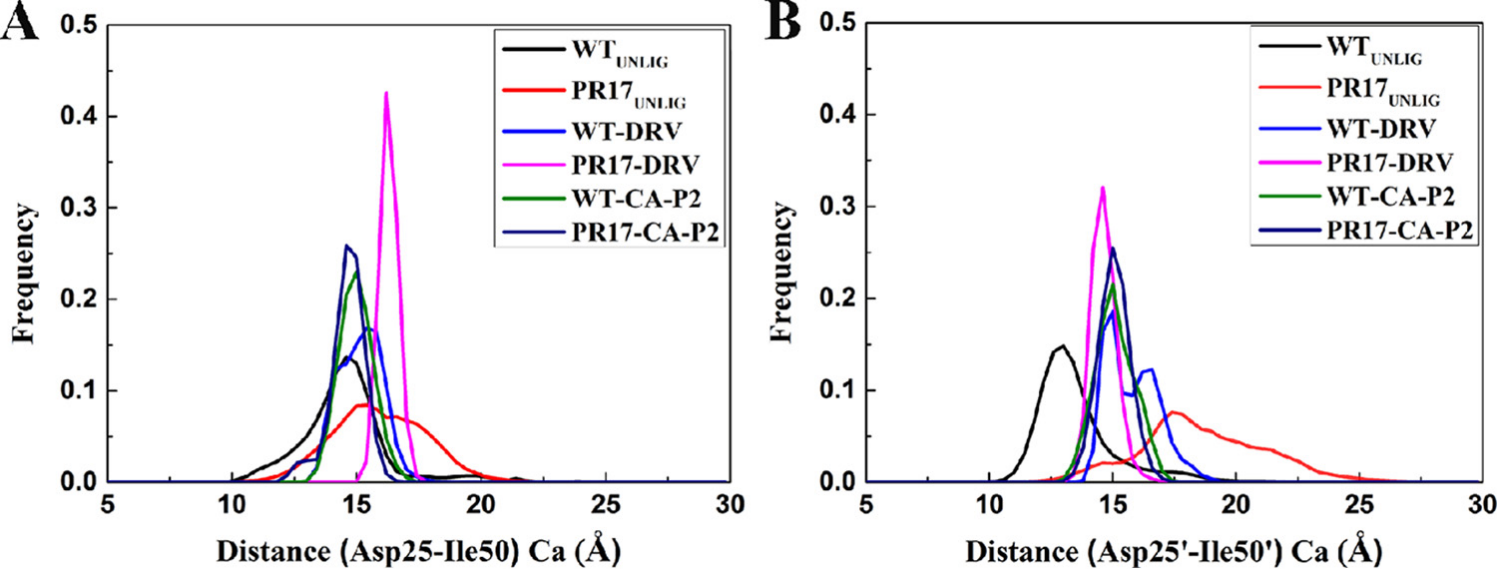
Histogram distributions of Asp25-Ile50 and Asp25’-Ile50’ distance in unligand PR and ligand bound PR complex.

The distance between aspartates (Asp25Ca and Asp25’Ca) and flap tips (Ile50Ca and Ile50’Ca) was also drawn. As seen from panel A of Fig. 3, for chain A of unligand WT and mutant PR, the distributions have a single peak near 15 Å. The average distances are 15.04 Å and 15.66 Å, respectively. For chain B, unligand WT and mutant PR have also one single sampling structure (panel B of Fig. 3). The average distances in WT PR and PR^S17^ are 12.74 Å and 17.73 Å, respectively. For chain A (Fig. 3A), the DRV-WT PR complex adopts two different sampling structures relative to other three complexes. For DRV-PR^S17^, CA-p2-WT PR, and CA-p2-PR^S17^ complexes, the distributions have a single peak approximately 16.2 Å, 14.9 Å, and 14.5 Å, respectively, while for DRV-WT PR, one peak is approximately 15.8 Å and the other peak is approximately 14.4 Å. The mean distances of Asp25-Ile50 for DRV-WT PR, DRV-PR^S17^, CA-p2-WT PR, and CA-p2-PR^S17^ complexes are 15.22 Å, 16.19 Å, 14.91 Å, and 14.27 Å, respectively. The DRV binding WT PR in chain B adopts two diverse conformational sampling as compared with other three complexes (Fig. 3B). For DRV-WT PR, the main peak is near 15 Å and the other minor peak is near 16.5 Å. While for DRV-PR^S17^, there is a peak around 14.6 Å, and CA-p2-WT PR and CA-p2-PR^S17^ complexes, the peaks are near 15 Å. The average distances in chain B for DRV-WT PR, DRV-PR^S17^, CA-p2-WT PR, and CA-p2-PR^S17^ complexes are 15.14 Å, 14.78 Å, 14.96 Å, and 15.07 Å, respectively. The distance of CA-p2-PR^S17^ complex is about 0.64 Å smaller than that of CA-p2-WT PR complex for chain A; oppositely in chain B the distance of CA-p2-PR^S17^ is about 0.11 Å larger than that in CA-p2-WT PR complex. However, the distance of DRV-PR^S17^ complex is about 0.97 Å larger than that of DRV-WT PR complex for chain A; oppositely in chain B the DRV-WT PR structures is about 0.36 Å smaller than DRV-WT PR complex. Therefore, the changes of the distance between the flap tips and the catalytic sites alter the volume of binding pocket, which must produce certain effect on the binding of ligands to PR.

**FIG 4:**
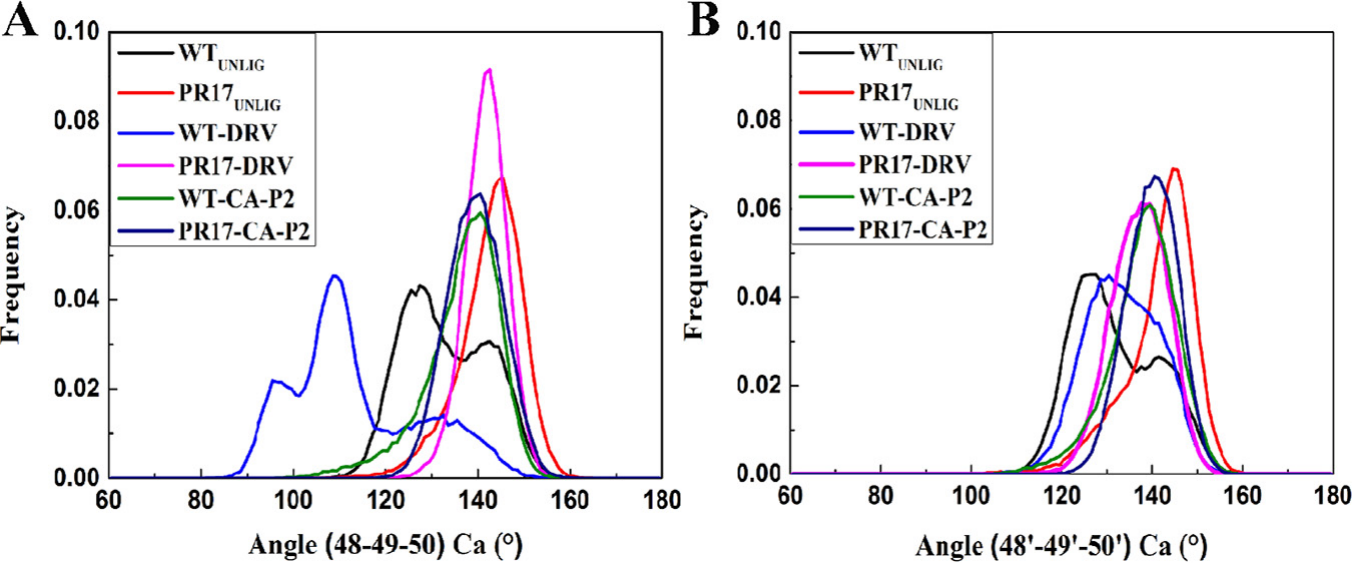
Histogram distributions for the TriCα angles (G48-G49-I50) and (G48’-G49’-I50’) in unligand PR and ligand bound PR complex.

TriCα in chain A (48Cα-G49Cα-I50Cα) and TriCα in chain B (48’Cα-G49’Cα-I50’Cα) were also analyzed (Fig. 4). For chain A, the mean values and standard deviation of TriCα(G48Cα-G490Cα-I50Cα) for unligand WT PR are 132.47° and 9.26°, respectively, whereas the mean values and standard deviation of TriCα (V48Cα-G490Cα-I50Cα) for unligand PR^S17^ are 141.13° and 7.01°, respectively. While for chain B, the TriCα(G48’Cα-G49’Cα-I50’Cα) mean values is 128.35° and standard deviation is 9.41 for unligand WT PR, whereas the TriCα(V48’Cα-G49’Cα-I50’Cα) mean values is 139.14° and standard deviation is 7.94° for unligand PR^S17^. As seen in Fig. 4A, the DRV-mutant, CA-p2-WT, and CA-p2-mutant PR complexes have a single peak near 140°; while the DRV-WT complex has two peaks, the main peak is near 109° and the other peak is near 98°. The mean values (the standard deviation) of TriCα angle in chain A for DRV-WT, DRV-mutant, CA-p2-WT, and CA-p2-mutant complexes are 109.96° (13.98°), 141.64° (4.64°), 138.36° (8.32°), and 139.39° (6.06°), respectively. As shown in Fig. 4B, for chain B, these four complexes have a single peak. The mean values of TriCα angle for DRV-WT, DRV-mutant, CA-p2-WT, and CA-p2-mutant complexes are 130.76° (8.18°), 138.13° (6.16°), 139.04° (7.60°), and 140.83° (5.67°), respectively. Hence, the mean values in chains A and B for DRV-PR^S17^ complex are larger than that of WT-PR complex, signifying the more curling of flap tips in DRV-PR^S17^ complex. The mean values in chains A and B for CA-p2-PR^S17^ complex are similar with that of WT PR complex, however, the standard deviation for CA-p2-R^S17^ complex are smaller than that of WT PR complex, implying that the small range of TriCα angles change of flap tips in CA-p2-PR^S17^ complex. Therefore, the flap region in DRV-PR^S17^ and CA-p2-PR^S17^ complexes has different mobility with that in WT complexes. On the basis of the key local fluctuations analysis for the PR complexes, we deduce that for unligand PRs and ligand-PR complexes, 17 mutated residues alter the flap-flap distance, the distance from flap regions to catalytic sites, the curling degree of flap tips, and the volume of binding pocket. For the unligand PR^S17^, the binding pocket exhibits an expansion phenomenon relative to other studied systems. These mutated residues increase the flexibility of flap region in PR^S17^-DRV complex; whereas they decrease the flexibility of the flap region in PR^S17^-CA-p2 complex.

### Binding free energy calculation.

To evaluate the binding abilities of DRV and CA-p2 to PRs, the various energy components were calculated applying MM-PBSA approach (Table 1). The binding energies of WT PR and PR^S17^ are estimated to be −14.15 and −8.71 kcal/mol when binding with DRV and −7.17 and −9.38 kcal/mol when binding with CA-p2, respectively. Compared with WT complex, the binding affinity of PR^S17^ with DRV decreases by 5.44 kcal/mol, while PR^S17^ with CA-p2 increased by 2.21 kcal/mol. This shows a similar trend compared with the experimental values of WT PR-DRV (−15.41 kcal/mol), PR^S17^-DRV (−9.95 kcal/mol), WT PR-CA-p2 (9.71 kcal/mol), and PR^S17^-CA-p2 (–10.44 kcal/mol) (20, 21).These results indicate the binding ability of the PR^S17^ with DRV is weakened, whereas its binding ability with CA-p2 is enhanced.

**TABLE 1:** Calculated binding free energy terms (kcal/mol) for DRV and CA-p2 binding to the WT and mutant HIV-1 protease by MM-PBSA method^*a*^

System	DRV-WT	DRV-mutant	CA-p2-WT	CA-p2-mutant
ΔE_vdW_	−67.12 ± 3.82	−60.82 ± 3.24	−70.83 ± 6.02	−75.32 ± 6.04
ΔE_ele_	−46.32 ± 4.19	−35.04 ± 4.22	−130.57 ± 21.14	−137.64 ± 24.29
ΔG_pb_	77.52 ± 5.05	64.80 ± 4.23	163.53 ± 7.76	172.32 ± 22.44
ΔG_SA_	−5.14 ± 0.14	−5.14 ± 0.10	−7.54 ± 0.32	−8.21 ± 0.35
ΔG_pol_	31.20	29.76	32.96	34.68
ΔG_nonpol_	−72.26	−65.96	−78.37	−83.53
ΔG_MM-PB/SA_	−41.06 ± 4.53	−36.20 ± 4.60	−45.41 ± 5.08	−48.85 ± 6.39
-TΔS	26.91 ± 2.63	27.49 ± 3.36	38.24 ± 3.17	39.47 ± 2.96
ΔG_bind_	−14.15 ± 5.64	−8.71 ± 5.69	−7.17 ± 5.99	−9.38 ± 7.04
ΔG_exp_	−15.41	−9.95	−9.71	−10.44

a ΔG_pol_ = ΔE_ele_ + ΔG_pb_; ΔG_nonpol_ = ΔE_vdW_ + ΔG_SA_; ΔG_MM-PB/SA_ = ΔE_ele_ + ΔE_vdW_ + ΔG_PB_ + ΔG_SA_; ΔG_bind_ = ΔG_MM-PB/SA_ − TΔS. The experimental binding free energies (ΔG_exp_) were derived from the experimental inhibition constants (K_i_) using the equation ΔG_exp_ = −RTlnK_i_.

As listed in Table 1, the favorable binding of these two ligands with PR comes from the van der Waals interactions (ΔE_vdW_), electrostatic interactions (ΔE_ele_), and nonpolar solvation energies (ΔG_SA_). However, polar solvation energies (ΔG_pb_) and entropies (-TΔS) are detrimental to the binding of PR to these two ligands. Compared with WT PR, the ΔE_ele_ of PR^S17^ with DRV is reduced by about 11.28 kcal/mol, and the ΔE_vdW_ is reduced by 6.30 kcal/mol, so the binding affinity is reduced. They make a major contribution to the resistance of PR^S17^ to DRV. For substrate CA-p2, except for the ΔG_pb_ and entropy, all other energy components are conducive to its binding with PR. Relative to the WT PR, ΔE_vdW_ and ΔE_ele_ in the energy of PR^S17^-CA-p2 increased by about −4.49 and −7.07 kcal/mol, respectively, and ΔG_pb_ and TΔS decreased by about 8.79 kcal/mol and 1.23 kcal/mol, respectively, therefore the binding affinity is enhanced.

The energies of DRV and CA-p2 to PRs were further computed applying SIE approach (Table 2). Relative to WT PR, the calculated energies of DRV and CA-p2 to PR^S17^ changed by 0.79 and −1.07 kcal/mol, respectively. PR^S17^ shows weak resistance toward DRV, but it enhances sensitivity to CA-p2. Compared with the WT PR, ΔE_vdW_ and intermolecular Coulomb interaction energies (ΔE_c_) of DRV and PR^S17^ are reduced by 4.94 and 1.30 kcal/mol, respectively, signifying the reduction of these two interactions provide a major contributions to resistance. The nonpolar interactions (ΔG_cav_) between DRV and PR^S17^ weakened slightly by 0.25 kcal/mol, which indicate that its reduction contributes little to the resistance. The ΔE_vdW_ and ΔE_c_ of CA-p2 with PR^S17^ increased by 0.08 and 10.67 kcal/mol, respectively, indicating that their increase helps enhance the energy with CA-p2. The ΔG_cav_ of CA-p2 with PR^S17^ also increased by 1.14 kcal/mol, indicating that its increase also helps enhance its binding with CA-p2. In summary, van der Waals interaction and electrostatic interaction are the main reasons that affect the binding of PR^S17^ with DRV or CA-p2. PR^S17^ shows weak resistance toward DRV, whereas its binding affinity to CA-p2 increased, indicating PR^S17^ enhances sensitivity to CA-p2.

**TABLE 2:** Calculated binding free energy terms (kcal/mol) for DRV and CA-p2 binding to the WT and mutant HIV-1 protease by SIE method^*a*^

System	DRV-WT	DRV-mutant	CA-p2-WT	CA-p2-mutant
ΔE_vdW_	−71.88 ± 3.24	−66.94 ± 3.32	−71.59 ± 2.99	−71.67 ± 2.98
ΔE_c_	−22.02 ± 2.72	−20.72 ± 1.91	−40.34 ± 2.14	−51.01 ± 2.14
ΔG_cav_	−12.22 ± 0.40	−11.97 ± 0.46	−15.36 ± 0.33	−16.50 ± 0.31
ΔG^R^	24.24 ± 2.56	25.29 ± 1.88	57.14 ± 1.92	58.79 ± 1.83
ΔG_pol_	2.22	5.57	16.80	7.78
ΔG_nonpol_	−84.10	−78.91	−86.95	−88.17
ΔG_bind_	−11.47 ± 0.43	−10.68 ± 0.41	−10.24 ± 0.37	−11.31 ± 0.36
ΔG_exp_	−15.41	−9.95	−9.71	−10.44

a ΔG_pol_ = ΔE_c_ + ΔG^R^; ΔG_nonpol_ = ΔE_vdW_ + ΔG_cav_. The experimental binding free energies (ΔG_exp_) were derived from the experimental inhibition constants (K_i_) using the equation ΔG_exp_ = −RTlnK_i_.

### Structure-binding affinity relationship analysis.

**FIG 5:**
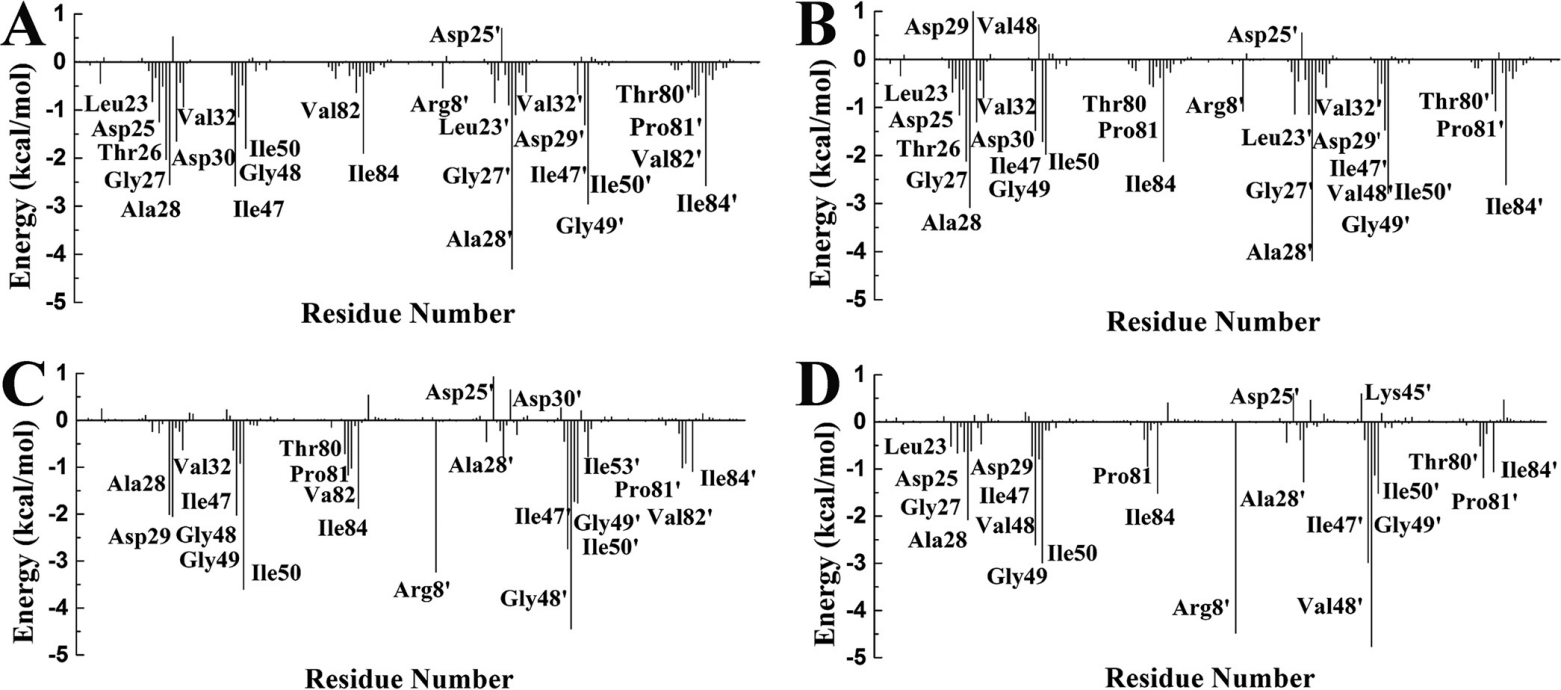
Free energy decomposition analysis for ligands with the mutant PR and the WT PR; residues contributing significantly are labled.

The ligand–residue interaction is used to study the reason for the change in binding affinity of PR^S17^ to DRV and CA-p2. Although there are some differences in the energy of importance residues, the interaction spectrum of each ligand to PR is very similar (Fig. 5 and Fig. S10). At least 12 residues around Ala28/Ala28’, Ile50/Ile50’, and Ile84/Ile84’ are favorable for the binding of PR and DRV or CA-p2. Each energy item is summarized in Tables S1 to S4. For the same ligand, the difference in energy (polar and nonpolar interaction) of PR is shown (Fig. S11 and S12). When binding with DRV and CA-p2, the nonpolar interaction of most of the important residues, Gly27/27’, Ala28/28’, Asp29/29’, Ile47/47’, Gly48/Val48’, Ile50/50’, Val82/Ser82’, and Ile84/84’, makes great contribution. However, the polar interaction of only a few residues, Gly27, Asp29, Asp30/30’, Ile47, Gly49, Gly48’, makes the significant contribution. Almost all of these residues have also been reported in the study by Weber et al. (20, 21). For example, the Leu group of CA-p2 can form multiple van der Waals interactions with residues Gly27, Ile50, Pro81’, Val82’, and Ile84’. The main-chain amide of Nle group in CA-p2 can form multiple hydrogen bonds with Asp29’ and Asp30’. And DRV can form multiple hydrogen bonds with residues Asp25’, Asp29’, and Asp30/Asp30’.

**FIG 6:**
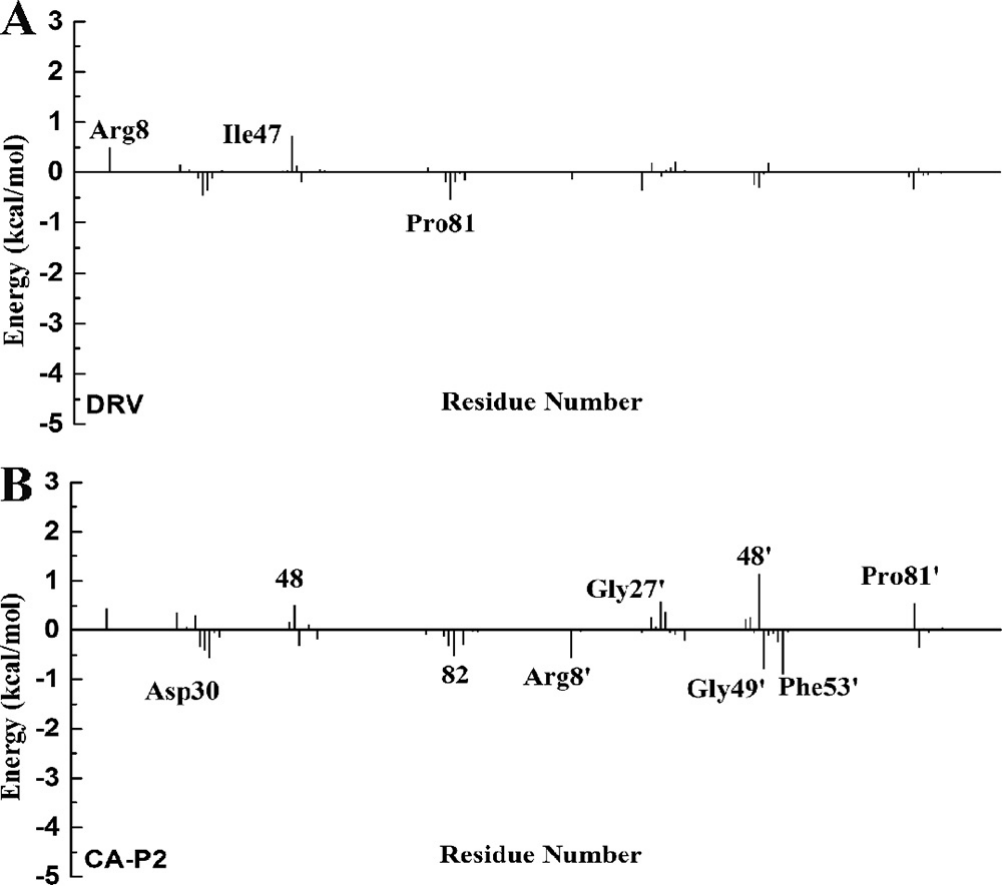
The difference of the nonpolar interaction (ΔG_vdW_ + ΔG_SA_) for two ligands with the mutant PR and the WT PR; residues contributing significantly are labled.

**FIG 7:**
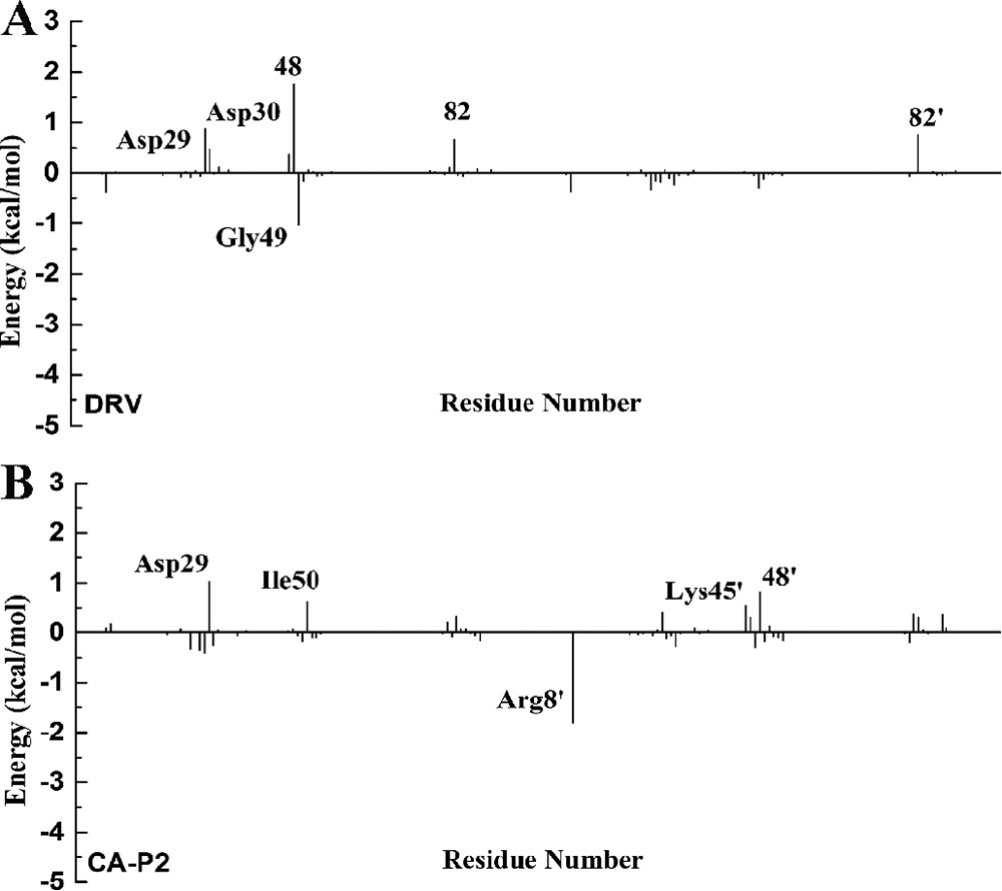
The difference of the polar interaction (ΔG_ele_ + ΔG_GB_) for two ligands with the mutant PR and the WT PR; residues contributing significantly are labled.

**FIG 8:**
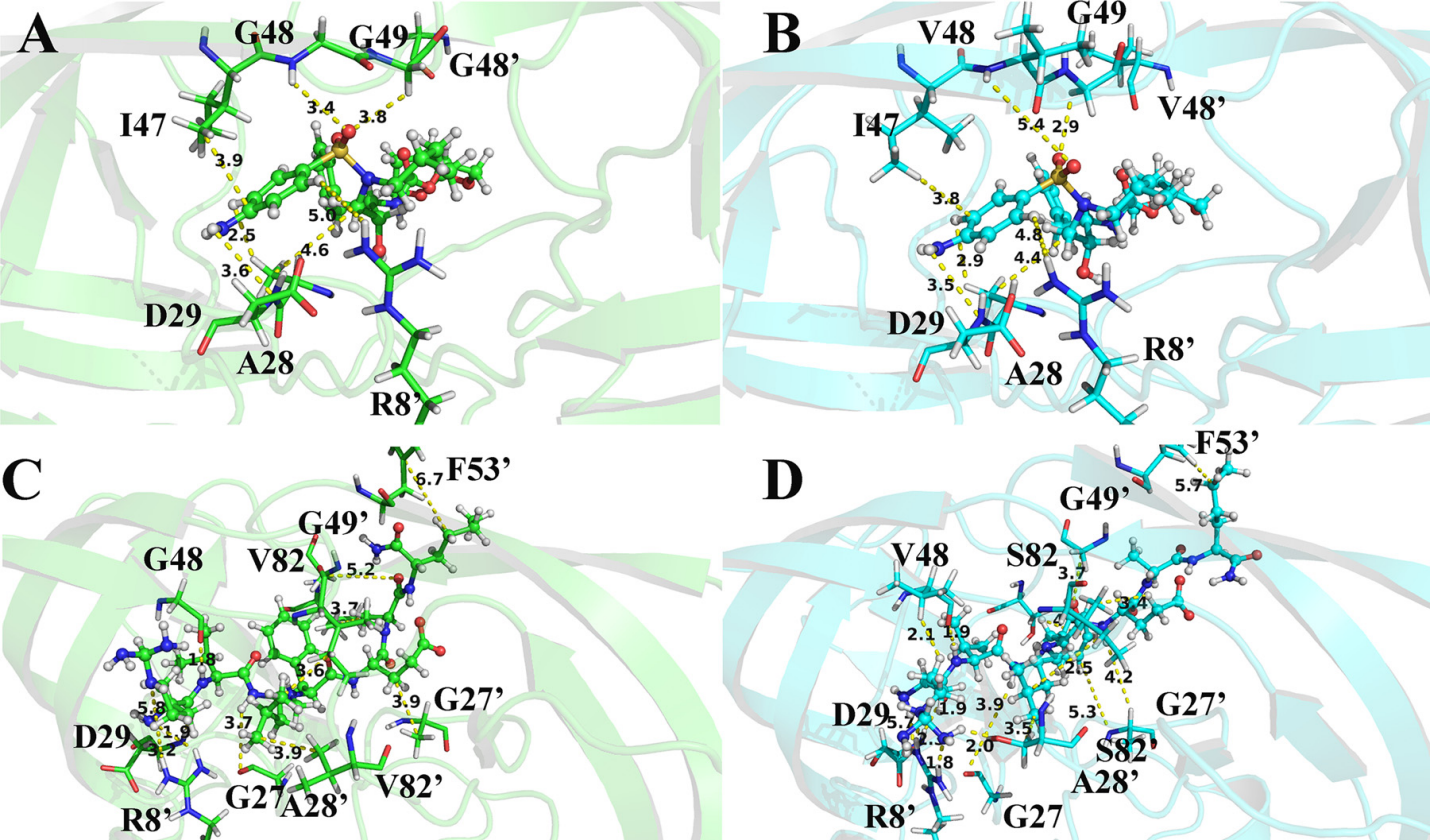
The represented MD structures of the ligands bound WT complexes and ligands bound mutant complexes: (A) the DRV bound PR; (B) the DRV bound PR^S17^; (C) the CA-P2 bound PR, (D) the CA-P2 bound PR^S17^. The represented structures extracted from the MD trajectories were used. The ligands are shown as ball-and-stick and the important residues are shown as sticks.

For the DRV-WT/mutant PR complexes, the total interaction energies of eight residues varies greatly (Fig. S10). Among them, four residues, Asp29, Ile47, 48, and 82’, in WT PR have stronger energy than those in PR^S17^, while the others (Ala28, Gly49, Arg8’, and 48’) in PR^S17^ form stronger interactions with DRV than those in WT PR (Tables S1 and S2). Comparing the difference of nonpolar interaction (Fig. 6), it can be seen there are three residues with an absolute difference larger than 0.5 kcal/mol, of which two residues (Arg8 and Ile47) reduce the binding of DRV and PR^S17^, while Pro81 is responsible increased binding of DRV and PR^S17^. The comparison of polar interaction (Fig. 7) shows that the absolute difference of six important residues is larger than 0.5 kcal/mol. Among these residues shown in Fig. 7, compared with WT complex, Gly49 forms stronger polar interactions, while Asp29, Asp30, 48, 82, and 82’ form weaker polar interactions in PR^S17^ complex. Relative to WT complex, the interaction between residue Ala28 in PR^S17^ and DRV is enhanced by 0.53 kcal/mol and van der Waals energy is increased by 0.46 kcal/mol. This is mainly caused by the reduced distance between the alkyl of Ala28 and hydrophobic group of DRV in PR^S17^ complex. The interactions between residue Gly49 and DRV in PR^S17^ is enhanced by 1.24 kcal/mol. As shown in Tables S1 and S2, electrostatic interaction of the backbone atoms for Gly49 in PR^S17^ with DRV is enhanced by 1.26 kcal/mol. This result is caused by the decrease of the distance between alkyl group of Gly49 and sulfonamide oxygen of DRV (Fig. 8). The interaction of between residue Arg8’ in PR^S17^ and DRV is enhanced by 0.54 kcal/mol. The distance between alkyl group of Arg8’ and aniline group of DRV in PR^S17^ complex decreased, which thus legitimately explained the increase of van der Waals energy (0.19 kcal/mol) in the interaction between Arg8’ and DRV. For residue 48’ in chain B, substituting valine for glycine resulted in an increase in the size of the hydrophobic side chain by an isopropyl group. This resulted in an increase in the interaction (0.62 kcal/mol) between V48’ in PR^S17^and DRV. However, the decrease in the interaction of residue 48 in chain A with DRV is mainly due to the decrease in electrostatic interaction (2.15 kcal/mol), which is the result of the increase in distance between DRV and Val48. This plays an important role in the loss of its energy in PR^S17^ complex. Mutated residues cause a reduction in electrostatic energy (1.26 kcal/mol) of the interaction between Asp29 and DRV. This is consistent with the reduction in the hydrogen bond occupancy between them. As shown in Fig. S10, the interaction of residue Ile47 in PR^S17^ to DRV is reduced. This is consistent with the increase in the distance between alkyl groups of Ile47 and hydrophobic group of DRV in PR^S17^ complex. For residue 82, substituting serine for valine causes the hydrophobic side chain to loss one methyl group. This can result in reduced interaction between Val82/Val82’ and DRV (0.49 and 0.84 kcal/mol). Therefore, we conclude that 17 mutated residues would distort the geometry of the binding pocket, leading to major conformational change of the key residues mentioned above (Fig. 8A and B), which would weaken the interactions between DRV and PR.

In the case of CA-p2-WT/mutant PR complexes, the total interaction energies of 10 residues has a large difference (Fig. S10). Among these 10 residues, residues Asp29, Gly49’, Ile50, Ile53’, and 82/82’ reduced the energy of PR^S17^ with CA-p2 by 1.44, 0.61, 0.62, 0.74, and 0.82/0.67 kcal/mol, respectively. These six residues play a major role in the loss of the binding ability of PR and CA-p2 resulted from the mutated residues. However, the interactions of Arg8’, Gly27, Ala28’, and 48 in PR^S17^ with CA-p2 increased by 1.24, 0.65, 0.50, and 0.59 kcal/mol, respectively (Tables S3 and S4). From Tables S3 and S4, and Fig. 6, there are nine residues whose absolute values of the difference in nonpolar interactions is greater than 0.50 kcal/mol, of which five residues (Arg8’, Asp30, Gly49’, Phe53’, and residue 82) have stronger nonpolar interactions and four residues (Gly27’, 48/48’, and Pro81’) have weaker nonpolar interactions with CA-p2 in PR^S17^ complex. Comparing the difference in polar interaction (Tables S3 and S4, Fig. 7) indicates that there are five major residues with absolute difference greater than 0.5 kcal/mol. Compared with WT PR complex, as shown in Fig. 7, the polar interactions formed by Arg8’ in PR^S17^ complex are stronger, while the polar interactions formed by Asp29, Lys45’, 48’, and Ile50 in PR^S17^ complex are weaker. The interaction between Arg8’ in PR^S17^ and CA-p2 is increased by 1.24 kcal/mol. These mutate residues enhance the polar energy of the interaction between Arg8’ and CA-p2, which is in consistent with the increase in the hydrogen bond occupancy between them (Table S5). The residue Gly27 in chain A in PR^S17^ has a strong interaction with CA-p2. The increase in binding energy between them mainly comes from the increase in electrostatic interaction energy (0.86 kcal/mol). This result is related to the high H-bond occupancy rate in PR^S17^ complex. The enhancement of the binding energy of Ala28’ in chain B and CA-p2 mainly due to the increased van der Waals energy (0.31 kcal/mol) and electrostatic interaction energy (0.44 kcal/mol). This can be seen from the decrease in the distance between them. The energy enhancement of V48 in PR^S17^ is mainly caused by the increase of van der Waals energy (0.51 kcal/mol). This energy change is related to the increase in side chain size caused by the G48V mutation and thus the high occupancy of H-bond. From the data of H-bond analysis (Table S5), it can be seen that atom O of CA-p2 forms a low-occupancy H-bond with the main chain amides of Asp29 in PR^S17^. This exactly explains the reduction in electrostatic energy (1.42 kcal/mol) in their interaction induced by the mutated residues. Furthermore, the decrease in van der Waals energy of Gly49’ and Ile50 with CA-p2 is the main reason for the reduction in binding energy between them. This is consistent with the increase in the C-H**^…^**π distance between central phenyl group in CA-p2 and alkyl groups of these two residues. The increase in the distance between the alkyl group of Ile53’ in PR^S17^ and the hydrophobic groups of CA-p2 reasonably explained the decrease of van der Waals interaction in their interaction induced by the mutated residues. For residue 82, replacing valine with serine brought about a decrease in the size of the hydrophobic side chain, which resulted in a decrease in the energy between Val82/Val82’ and CA-p2. In addition, compared with WT PR complex, the position of 80 loop in PR^S17^ shifts to the active pocket, leading to the increase of van der Waals contacts of residues Pro81’ and Ile84’ with Leu group in CA-p2 (Fig. S13). Weber et al. also showed by X-ray diffraction that the 80's loop (residues 80' to 85') has a similar movement, which is beneficial to increase the binding affinity of PR to CA-p2 (20). Significantly, as can be seen from Table S5, relatively to WT complex, the occupancy rate of the two hydrogen bonds formed by nitrogen atom of the guanidine group in CA-p2 and Arg8' in PR^S17^ complex is increased. And the nitrogen atom of the guanidine group also forms new hydrogen bonds with Pro81' and Ser82' in PR^S17^ complex. These enhanced interactions of the guanidine group in CA-p2 with PR^S17^ play an important role in the increase of its binding ability. Therefore, when designing inhibitors in the future, it can be a good choice to introduce of polar groups similar to guanidine group. Briefly, the 17 mutated residues trigger the structural change of the above-mentioned residues in the binding site, thereby enhancing the interactions between CA-p2 and PR.

From the above analysis of DRV and CA-p2 with PRs, it can be seen that 17 mutated residues caused significant changes in flap dynamics and active site movements, thereby changing the interactions between residues (around Ala28/Ala28’, Ile50/Ile50’, and Ile84/Ile84’) and DRV and CA-p2 in PR^S17^ complex. And most noticeable of all, in the CA-p2-PR^S17^ complex, the mutated residues not only caused the increase of the original hydrogen bond occupancy between the guanidine group in CA-p2 and Arg8', but also make the nitrogen atom of guanidine group form new hydrogen bonds with Pro81' and Ser82, which helps increase the binding ability between CA-p2 and PR^S17^.

### Conclusions.

Focused on the structure of unligand PRs and PR-ligand complexes, we executed multiple MD simulations to probe the local structural changes and the difference in sensitivity of PR^S17^ to DRV and CA-p2 due to 17 mutated residues. Furthermore, MM-PBSA and SIE were used to research the detailed interaction between PR and these two ligands.

The simulations results demonstrate that 17 mutated residues trigger the spatial redistribution of their surrounding residues, and further affect the active site movements, thereby changing the interaction of these residues with DRV and CA-p2 in variant PR^S17^. For the unligand variant PR^S17^, the binding pocket exhibits an expansion phenomenon relative to other studied WT PR and PR-complexes. Moreover, free energy analysis indicate that the residues (around Ala28/Ala28’, Ile50/Ile50’, and Ile84/Ile84’) in hydrophobic cavity make an important contribution to the binding of PR and ligands. It is particularly pointed out that the enhancement of the interaction energy between the guanidine group of CA-p2 and Arg8' of PR^S17^ is the main reason for the increase of its binding affinity. Based on our results, we can suggest two factors that may be helpful to design effective inhibitors targeting HIV-1 PR: (i) importance of hydrophobic cavity for binding and sensitivity, and (ii) introduction of polar groups similar to the guanidine group.

These finding clarify the details of DRV and CA-p2 bound to PR which will help design novel drugs targeting HIV-1 protease.

## MATERIALS AND METHODS

### Preparation of the unligand PR and the PR-ligands complexes.

The initial models were taken from protein data bank: 4DQB for DRV-WT PR complex (46), 5T2Z for DRV-PR^S17^ complex (21), 6O48 for the CA-p2-WT PR complex (20), 6O5X for the CA-p2-PR^S17^ complex (20), 1HHP for the WT PR (45), 5T2E for the variant PR^S17^ (21). Our methods include (i) four MD simulations for DRV-PR and CA-p2-PR complexes to study their interactions between PR and ligand, (ii) two MD simulations of unligand PR (WT PR and variant PR^S17^) to compare the structure changes of PR, (iii) four MM-PBSA calculations to compute the binding free energy, and (iv) four SIE calculations to further validate the results of MM-PBSA. Considering the importance of the protonation of Asp25 in chain B, a proton is added to its oxygen atom OD2 (47, 48). All water molecules were kept in initial modes. Using LEaP module, the missing atoms were added (49). The parameters of PR and ligands (DRV and CA-p2) was generated using AMBER ff03 force filed and GAFF with AM1-BCC charges (50), respectively. Explicit solvation has been represented by the TIP3P water model (51), and the truncated octahedral periodic boundary conditions have been applied using the cutoff distance 12 Å from the solute to the edge of box containing more than 10,000 water molecules. To neutralize the charge of simulation systems, six chloride counterions for WT PR complexes, two chloride counterions for the variant PR^S17^ complexes, and three chloride counterions for unligand PR were added.

### Molecular dynamics simulation of the unligand PRs and the PR-ligands complexes.

Amber12 package were used to execute the energy minimization and MD simulation. To eliminate any bad interatomic contacts, the initial system with weak restraint was minimized based on the steepest descent method of 2,000 steps followed by the conjugate gradient method of 6,000 steps, and then all atoms are minimized by 8,000 steps without restriction. Subsequently, under the 600 ps simulation, 10 kcal mol^−1 ^Å^−2^ restraints was placed on all solute atoms, each system was slowly heated from 0 to 310 K. After that, the density was equilibrated for 800 ps when 2 ps^−1^ coupling constant was used for the Berendsen barostat. Finally, under an isothermal isobaric ensembles (NPT) ensemble, using a Langevin dynamics temperature scaling with collision frequency 2 ps^−1^, production MD simulations were executed. To give the reasonable results for each system, three repeated 250 ns simulations were carried out. According to the Maxwellian distribution, the initial velocities were set, and random seeds with three different values were assigned to these repeat simulations. A single trajectory is obtained from the equilibrated trajectories of three repeated simulations for post-processing analysis. During MD simulations, particle mesh Ewald (PME) method (52) was used to treat long-range electrostatic. The SHAKE (53) algorithm was used to restrain all bonds. For van der Waals and long-range electrostatic interactions, the cutoff distances are set to 12 Å. Visualizing the trajectories and depicting structural representations were done using PyMol software (54).

### Calculation of molecular mechanics Poisson-Boltzmann surface area.

When applying MM-PBSA method, 1,000 snapshots structures at 600 ps intervals were extracted from the equilibrated trajectories of the joined simulations. The binding affinity is computed from energies of PR, ligands (DRV and CA-p2), and complex:

(1) $$\Delta G_{\mathrm{bind}}=G_{\mathrm{complex}}-G_{\mathrm{PR}}-G_{\mathrm{ligand}}$$

G_complex_, G_PR_, and G_ligand_ are the energies of complex, PR, and ligands, respectively. They can be computed as follows:

(2) $$G_{X}=E_{\mathrm{MM}}+E_{\mathrm{sol}}-\mathrm{TS}$$

Here, G is divided into molecular mechanics energy (E_MM_), the solvation energy (E_sol_), and the conformational entropy (TS).

(3) $$E_{\mathrm{MM}}=E_{\mathrm{int}}+E_{\mathrm{ele}}+E_{\mathrm{vdW}}$$

Where, E_MM_ consists of internal energy (E_int_), electrostatic energies component (E_ele_), and van der Waals energy component (E_vdW_). E_sol_ is composed of polar solvation energy (G_pb_) and nonpolar solvation energy (G_SA_):

(4) $$E_{\mathrm{solv}}=G_{\mathrm{pb}}+G_{\mathrm{SA}}$$

G_pb_ was estimated with PBSA module. In MM-PBSA calculation for polar solvation energy, 80 and 1 were used for the exterior dielectric constant and the solute dielectric constant, respectively (55). And the ionic strength was set to 0.1 M. G_SA_ was estimated as a function of solvent-accessible surface area (SASA):

(5) $$G_{\mathrm{SA}}=\gamma \mathrm{SASA}+\beta$$

SASA is determined with a probe radius of 1.4 Å. Values *γ* and *β* were set to 0.00542 kcal mol^−1 ^Å^−2^ and 0.92 kcal mol^−1^, respectively (56). In addition, TS was calculated with normal-mode analysis. Considering the high computational demand, only 100 snapshots for each system were used to compute the entropy.

Because PB calculations require high computational demand, MM/GBSA method was used to the energy decomposition. The energy contribution can be assigned to each residue from the association of PR with DRV and CA-p2, including four energy terms: E_vdW_, E_ele_, E_GB_, and G_SA_.

### Calculation of solvated interaction energy method.

SIE is an energy prediction approach based on empirical equations (43, 57, 58). The same 1,000 snapshot structures as in MM-PBSA were used for SIE analyses. The energy between PR and ligand was computed as following:

(6) $$\Delta G_{\mathrm{bind}}\left(\rho ,D_{\mathrm{in}},\alpha ,\gamma ,C\right)=\alpha \left[E_{\mathrm{Coul}}\left(D_{\mathrm{in}}\right)+\Delta G^{R}\left(\rho ,D_{\mathrm{in}}\right)+E_{\mathrm{vdW}}+\Delta G_{\mathrm{cav}}\left(\rho \right)\right]+C$$

E_Coul_ and E_vdW_ are the intermolecular Coulomb and vdW interaction, respectively. ΔG^R^ indicates the change of the reaction energy (59). ΔG_cav_ indicates the change of the nonpolar solvation energy. ΔG_cav_ can be estimated as a function of change in the molecular surface area (ΔMSA):

(7) $$\Delta G_{\mathrm{cav}}=\gamma \Delta \mathrm{MSA}$$

The above parameters are optimized by fitting to the experimental energies. The values are *ρ =* 1.1, D_in_ = 2.25, α = 0.1048, γ = 0.0129 kcal mol^−1 ^Å^−2^, and C = −2.89 kcal mol^−1^. The Sietraj was used to run SIE calculations (43).

### Analysis of the conformational dynamics for PR.

To explore the impact of 17 mutated residues on the internal dynamics of PR, the cross-correlation matrix (C_ij_) was calculated (60), as follows:

(8) $$C_{\mathrm{ij}}=\frac{<\Delta r_{i}\Delta r_{j}>}{{\left(<\Delta r_{i}^{2}><\Delta r_{j}^{2}>\right)}^{2}}$$

angle brackets denote the time average of MD simulation, Δr_i_ denotes the displacement vectors of the average position of Cα atom in i^th^ residue. The matrix (C_ij_) fluctuates between −1.0 and 1.0. The positive value and negative value of C_ij_ indicate the positive and anticorrelated movement of residue i with respect to residue j, respectively.
