# Supplementary material for: Multiple Molecular Dynamics Simulations and Energy Analysis Unravel the Dynamic Properties and Binding Mechanism of Mutants HIV-1 Protease with DRV and CA-p2
Source: Microbiol Spectr. 2022 Mar 23;10(2):e00748-21. doi: 10.1128/spectrum.00748-21 (PMC9045218; doi:10.1128/spectrum.00748-21)
Supplement: SUPPLEMENTAL FILE 1 — Supplemental material. Download SPECTRUM00748-21_Supp_1_seq4.pdf, PDF file, 2.9 MB [file spectrum00748-21_supp_1_seq4.pdf]

Supporting Information

Multiple Molecular Dynamics Simulations and Energy Analysis Unravel the Dynamic Properties and Binding Mechanism of Mutants HIV-1 Protease with DRV and CA-p2

Ruige Wang, Qingchuan Zheng *

Institute of Theoretical Chemistry, College of Chemistry, Jilin University, 2 Liutiao Road, Changchun 130023, P. R. China.

Corresponding Author

Qing-Chuan Zheng: Tel: +86-431-88498966; Fax: +86-431-88498966

E-mail: zhengqc@jlu.edu.cn

Table of contents

Table S1. Relative binding free energies (kcal/mol) contributed by important residues for the WT PR-DRV complex.

Table S2. Relative binding free energies (kcal/mol) contributed by important residues for the mutant PR-DRV complex.

Table S3. Relative binding free energies (kcal/mol) contributed by important residues for the WT PR-CA-p2 complex.

Table S4. Relative binding free energies (kcal/mol) contributed by important residues for the mutant PR-CA-p2 complex.

Table S5. Hydrogen bonds formed by two ligands DRV and CA-p2 with the WT and mutated HIV-1 PR.

Fig. S1. Time evolution of the RMSD values relative to the initial structure in ligand bound WT PR complexes and ligand bound mutant PR complexes.

Fig. S2. Time evolution of the RMSD values relative to the initial structure of unligand PR.

Fig. S3. The initial conformation and the representative conformation extracted by clustering analysis for the unligand PR from the MD simulations: (A) unligand WT PR; (B) unligand PR^{S17}.

Fig. S4. The fluctuations and the accumulated mean values of enthalpies for the ligand bound WT PR complexes and ligand bound mutant PR complexes.

Fig. S5. The fluctuations and the accumulated mean values of entropies for the ligand bound WT PR complexes and ligand bound mutant PR complexes.

Fig. S6. RMSF of the Cα atoms versus residue number of the HIV-1 PR in the research

systems.

Fig. S7. Cross-correlation matrices of the fluctuations of the coordinates for Cα atoms near their corresponding average positions of the 600 ns MD simulations: (A) unligand WT PR; (B) unligand PRS17; (C) DRV-WT PR; (D) DRV-PRS17; (E) CA-p2-WT PR; (F) CA-p2-PRS17.

Fig. S8. Contour maps of the free energy as a function of the backbone angle φ and ψ. I50 and I50' describe the residues of the chain A and B in the PR, respectively. (A) unligand WT PR; (B) unligand PR^{S17}; (C) DRV-WT PR; (D) DRV-PR^{S17}; (E)CA-p2-WT PR; (F) CA-p2-PR^{S17}.

Fig. S9. Superimposed the structure of clustering analysis for the WT PR and variant PR^{S17}. The WT PR and variant PR^{S17} are shown in green and cyan, respectively. A. unligand PR; B. the PR-DRV complex; C. The PR-CA-p2 complex.

Fig. S10. The difference between the interaction enthalpy of ligands DRV and CA-p2 with the mutated and WT PR.

Fig. S11. The nopolar interaction energy of per-residue basis for the mutated and WT PR with ligands complexes.

Fig. S12. The polar interaction energy of per-residue basis for the mutated and WT PR with ligand complexes.

Fig. S13. Superimposed the structure of clustering analysis for the WT PR and variant PR^{S17} complexed with CA-p2. The WT PR and variant PR^{S17} are shown in green and cyan, respectively.

Table S1. Relative binding free energies (kcal/mol) contributed by important residues for the WT PR-DRV complex.

Residue	S_{vdW}	B_{vdW}	T_{vdW}	S_{ele}	B_{ele}	T_{ele}	S_{GB}	B_{GB}	T_{GB}	S_{SUR}	B_{SUR}	T_{SUR}	T_{GBTOT}
Leu23	-0.41	-0.04	-0.45	0.04	-0.11	-0.06	-0.26	-0.03	-0.29	-0.03	0.00	-0.03	-0.83
Asp25	-1.06	0.00	-1.06	1.03	0.00	1.03	-1.18	0.00	-1.18	-0.05	0.00	-0.05	-1.26
Thr26	-0.03	-0.11	-0.14	-0.01	0.07	0.06	0.19	-0.62	-0.43	0.00	0.00	0.00	-0.51
Gly27	0.00	-0.72	-0.72	0.00	-0.21	-0.21	0.00	-1.04	-1.04	0.00	-0.07	-0.07	-2.04
Ala28	-0.57	-1.26	-1.82	0.12	-0.30	-0.18	0.03	-0.41	-0.38	-0.08	-0.1	-0.18	-2.56
Asp29	-0.35	-0.52	-0.87	-0.94	-0.20	-1.14	2.13	0.51	2.64	-0.07	-0.04	-0.11	0.53
Asp30	-0.24	-0.15	-0.38	-2.80	-3.36	-6.16	2.84	2.10	4.94	-0.02	-0.04	-0.06	-1.66
Val32	-0.44	-0.10	-0.53	0.19	-0.31	-0.13	-0.41	0.15	-0.26	-0.02	0.00	-0.02	-0.94
Ile47	-1.24	-0.32	-1.55	-0.37	-0.54	-0.91	-0.08	-0.01	0.06	-0.19	0.00	-0.19	-2.59
Gly48	0.00	-0.98	-0.98	0.00	-1.23	-1.23	0.00	1.18	1.18	0.00	-0.12	-0.12	-1.15
Gly49	0.00	-0.80	-0.80	0.00	-0.23	-0.23	0.00	0.63	0.63	0.00	-0.09	-0.09	-0.48
Ile50	-1.30	-0.34	-1.65	-0.24	0.40	0.16	0.10	-0.22	-0.12	-0.19	-0.01	-0.20	-1.80
Pro81	-0.16	-0.06	-0.22	-0.19	0.11	-0.08	0.12	0.06	0.17	-0.02	0.00	-0.02	-0.15
Val82	-0.40	-0.11	-0.50	-0.00	0.06	0.06	-0.15	0.03	-0.11	-0.09	-0.00	-0.09	-0.64
Ile84	-1.37	-0.10	-1.47	0.07	-0.26	-0.19	-0.29	0.18	-0.11	-0.13	-0.00	-0.13	-1.91
Arg8’	-0.14	-0.01	-0.15	0.40	0.01	0.41	-0.70	-0.08	-0.78	-0.02	-0.00	-0.02	-0.55
Leu23’	-0.38	-0.05	-0.43	0.04	-0.11	-0.07	-0.31	-0.01	-0.32	-0.04	-0.00	-0.04	-0.86
Asp25’	-0.12	-0.17	-0.29	-13.73	0.09	-13.64	15.02	-0.28	14.74	-0.11	-0.00	-0.11	0.71
Gly27’	0.00	-0.93	-0.93	0.00	0.97	0.97	0.00	-0.87	-0.87	0.00	-0.06	-0.06	-0.90
Ala28’	-0.59	-1.46	-2.04	0.15	-1.94	-1.79	-0.02	-0.34	-0.36	-0.04	-0.08	-0.12	-4.31
Asp29’	-0.67	-0.60	-1.27	2.05	-1.72	0.33	-0.85	0.80	-0.05	-0.06	-0.05	-0.11	-1.10
Val32’	-0.41	-0.11	-0.52	0.13	-0.13	0.01	-0.28	0.19	-0.10	-0.03	0.00	-0.03	-0.64
Ile47’	-0.52	-0.10	-0.62	0.05	0.16	0.21	-0.05	-0.14	-0.19	-0.08	0.00	-0.08	-0.68
Gly48’	0.00	-0.58	-0.58	0.00	-0.71	-0.71	0.00	1.53	1.53	0.00	-0.14	-0.14	0.11
Gly49’	0.00	-0.80	-0.80	0.00	-1.34	-1.34	0.00	0.95	0.95	0.00	-0.12	-0.12	-1.31
Ile50’	-1.72	-0.57	-2.29	-0.55	-0.52	-1.06	0.27	0.37	0.63	-0.19	-0.05	-0.24	-2.96
Thr80’	-0.24	-0.09	-0.32	0.00	-0.02	-0.01	-0.03	-0.20	-0.23	-0.01	0.00	-0.01	-0.57
Pro81’	-0.47	-0.17	-0.63	-0.16	0.07	-0.09	0.08	-0.04	0.04	-0.06	0.00	-0.06	-0.74
Val82’	-0.45	-0.31	-0.76	0.04	-0.13	-0.09	-0.14	0.40	0.26	-0.11	0.00	-0.11	-0.70
Ile84’	-1.85	-0.14	-1.99	0.16	-0.02	0.14	-0.34	-0.17	-0.51	-0.21	0.00	-0.21	-2.58

Table S2. Relative binding free energies (kcal/mol) contributed by important residues for the mutant PR-DRV complex.

Residue	S_{vdW}	B_{vdW}	T_{vdW}	S_{ele}	B_{ele}	T_{ele}	S_{GB}	B_{GB}	T_{GB}	S_{SUR}	B_{SUR}	T_{SUR}	T_{GBTOT}
Leu23	-0.27	-0.04	-0.31	0.05	-0.13	-0.08	-0.27	-0.01	-0.28	-0.02	0.00	-0.02	-0.69
Asp25	-1.01	0.00	-1.01	1.12	0.00	1.12	-1.24	0.00	-1.24	-0.04	0.00	-0.04	-1.17
Thr26	-0.04	-0.13	-0.17	0.01	-0.02	-0.01	0.21	-0.67	-0.46	0.00	0.00	0.00	-0.63
Gly27	0.00	-0.85	-0.85	0.00	-0.94	-0.94	0.00	-0.27	-0.27	0.00	-0.07	-0.07	-2.12
Ala28	-0.78	-1.50	-2.28	0.11	-0.34	-0.23	0.03	-0.42	-0.40	-0.10	0.09	-0.19	-3.09
Asp29	-0.54	-0.67	-1.21	-1.85	-0.06	-1.92	3.86	0.44	4.30	-0.10	-0.04	-0.13	1.04
Asp30	-0.24	-0.26	-0.51	-3.42	-2.93	-6.35	3.51	2.10	5.61	-0.03	-0.03	-0.06	-1.31
Val32	-0.41	-0.10	-0.51	0.27	-0.33	-0.05	-0.49	0.28	-0.21	-0.02	0.00	-0.02	-0.80
Ile47	-0.75	-0.12	-0.87	-0.10	-0.10	-0.20	-0.09	-0.20	-0.28	-0.14	0.00	-0.14	-1.49
Val48	-0.15	-0.71	-0.85	-0.41	1.34	0.92	0.05	0.73	0.78	-0.01	-0.11	-0.12	0.73
Gly49	0.00	-0.99	-0.99	0.00	-1.49	-1.49	0.00	0.85	0.85	0.00	-0.09	-0.09	-1.72
Ile50	-1.18	-0.51	-1.70	-0.47	0.33	-0.15	0.20	-0.16	0.03	-0.14	-0.03	-0.17	-1.98
Thr80	-0.23	-0.09	-0.32	0.05	-0.02	0.03	0.09	-0.31	-0.23	-0.01	0.00	-0.01	-0.52
Pro81	-0.47	-0.23	-0.69	0.01	0.04	0.05	-0.01	0.17	0.16	-0.09	0.00	-0.09	-0.57
Ser82	-0.33	-0.36	-0.69	-0.06	0.03	-0.03	0.41	0.23	0.65	-0.08	-0.01	-0.09	-0.15
Ile84	-1.50	-0.12	-1.61	0.08	-0.26	-0.18	-0.36	0.17	-0.19	-0.15	0.00	-0.15	-2.13
Arg8’	-0.28	-0.01	-0.29	0.93	0.01	0.94	-1.57	-0.13	-1.70	-0.03	0.00	-0.03	-1.09
Leu23’	-0.67	-0.07	-0.74	0.07	-0.05	0.03	-0.28	-0.07	-0.35	-0.08	0.00	-0.08	-1.14
Asp25’	0.06	-0.18	-0.12	-11.17	0.17	-11.00	12.07	-0.31	11.76	-0.08	0.00	-0.08	0.56
Gly27’	0.00	-0.98	-0.98	0.00	1.06	1.06	0.00	-1.15	-1.15	0.00	-0.09	-0.09	-1.15
Ala28’	-0.58	-1.42	-2.00	0.17	-2.10	-1.93	-0.00	-0.15	-0.15	-0.04	-0.08	-0.12	-4.20
Asp29’	-0.61	-0.57	-1.18	2.33	-1.64	0.69	-1.31	0.79	-0.52	-0.07	-0.05	-0.11	-1.12
Val32’	-0.39	-0.11	-0.49	0.04	-0.06	-0.02	-0.23	0.18	-0.06	-0.02	0.00	-0.02	-0.59
Ile47’	-0.77	-0.10	-0.87	0.02	0.22	0.23	-0.04	-0.24	-0.27	-0.08	0.00	-0.08	-0.99
Val48’	-0.33	-0.54	-0.87	-0.02	-0.77	-0.79	-0.09	1.39	1.30	-0.06	-009	-0.15	-0.51
Gly49’	0.00	-0.87	-0.87	0.00	-1.31	-1.31	0.00	0.80	0.80	0.00	-0.09	-0.09	-1.47
Ile50’	-1.55	-0.59	-2.14	-0.38	-0.71	-1.09	0.19	0.45	0.64	-0.18	-0.04	-0.21	-2.80
Thr80’	-0.30	-0.12	-0.41	0.18	-0.17	0.01	-0.20	-0.11	-0.31	-0.01	0.00	-0.01	-0.73
Pro81’	-0.65	-0.30	-0.95	-0.72	0.33	-0.39	0.50	-0.15	0.35	-0.09	0.00	-0.09	-1.08
Ser82’	-0.20	-0.51	-0.72	0.04	0.02	0.07	0.44	0.43	0.87	-0.07	-0.01	-0.08	0.14
Ile84’	-1.88	-0.18	-2.05	0.09	0.02	0.11	-0.26	-0.22	-0.47	-0.20	0.00	-0.20	-2.62

Table S3. Relative binding free energies (kcal/mol) contributed by important residues for the WT PR-CA-p2 complex.

Residue	S_{vdW}	B_{vdW}	T_{vdW}	S_{ele}	B_{ele}	T_{ele}	S_{GB}	B_{GB}	T_{GB}	S_{SUR}	B_{SUR}	T_{SUR}	T_{GBTOT}
Gly27	0.00	-0.57	-0.57	0.00	-0.07	-0.07	0.00	0.71	0.71	0.00	-0.06	-0.06	0.01
Ala28	-0.54	-0.97	-1.51	0.06	-1.62	-1.56	-0.09	1.31	1.22	-0.10	-0.08	-0.17	-2.02
Asp29	-0.92	-0.36	-1.28	7.57	-0.75	6.82	-6.92	-0.46	-7.38	0.16	-0.05	-0.21	-2.06
Val32	-0.55	-0.12	-0.68	-1.15	1.42	0.27	1.23	-1.42	-0.19	-0.04	0.00	-0.04	-0.63
Ile47	-0.75	-0.30	-1.05	-0.70	1.05	0.36	0.89	-0.72	0.17	-0.12	0.00	-0.12	-0.64
Gly48	0.00	-1.41	-1.41	0.00	-3.40	-3.40	0.00	3.11	3.11	0.00	-0.32	-0.32	-2.03
Gly49	0.00	-1.69	-1.69	0.00	-2.53	-2.53	0.00	3.46	3.46	0.00	-0.17	-0.17	-0.92
Ile50	-2.25	-0.89	-3.13	-2.19	1.10	-1.09	2.23	-1.31	0.92	-0.28	-0.03	-0.31	-3.61
Thr80	-0.28	-0.15	-0.44	0.46	0.15	-0.31	0.09	-0.04	0.05	-0.02	0.00	-0.02	-0.72
Pro81	-0.98	-0.35	-1.33	-3.86	3.34	-0.52	4.18	-3.27	0.91	-0.23	0.00	-0.23	-1.17
Val82	-0.86	-0.41	-1.27	-1.96	2.16	0.19	2.05	-1.79	0.26	-0.20	0.00	-0.20	-1.03
Ile84	-1.50	-0.14	-1.64	-1.35	0.99	-0.37	1.29	-0.97	0.32	-0.20	0.00	-0.20	-1.89
Arg87	-0.11	-0.03	-0.14	-9.57	0.03	-9.54	10.12	0.10	10.22	0.00	0.00	0.00	0.54
Arg8’	-0.62	-0.03	-0.64	-16.61	0.04	-16.57	14.26	0.06	14.32	-0.35	0.00	-0.35	-3.24
Asp25’	-0.54	-0.07	-0.61	12.10	1.90	13.99	-10.50	-1.87	-12.37	-0.08	0.00	-0.08	0.94
Ala28’	-0.32	-0.47	-0.79	0.13	-1.70	-1.57	-0.16	1.88	1.72	-0.09	-0.06	-0.15	-0.78
Asp30’	-0.23	-0.16	-0.39	20.37	3.00	23.37	-19.44	-2.79	-22.23	-0.08	-0.02	-0.10	0.66
Ile47’	-1.09	-1.21	-2.30	-2.60	-0.66	-3.26	2.58	0.43	3.01	-0.14	-0.05	-0.19	-2.75
Gly48’	0.00	-1.79	-1.79	0.00	-4.08	-4.08	0.00	1.74	1.74	0.00	-0.32	-0.32	-4.45
Gly49’	0.00	-1.71	-1.71	0.00	-1.62	-1.62	0.00	1.72	1.72	0.00	-0.13	-0.13	-1.74
Ile50’	-1.14	-0.68	-1.82	-1.53	0.30	-1.23	1.63	-0.18	1.45	-0.13	-0.04	-0.17	-1.77
Phe53’	-0.84	-0.20	-1.04	-0.47	-0.51	-0.98	0.69	0.74	1.43	-0.19	0.00	-0.19	-0.78
Pro81’	-0.74	-0.35	-1.09	-1.77	1.62	-0.15	1.98	-1.54	0.44	-0.20	-0.02	-0.22	-1.02
Val82’	-0.81	-0.36	-1.17	-0.92	1.15	0.23	1.14	-0.94	0.21	-0.19	0.00	-0.19	-0.93
Ile84’	-0.93	-0.09	-1.01	-1.07	1.30	0.23	1.16	-1.30	-0.13	-0.17	0.00	-0.17	-1.00

Table S4. Relative binding free energies (kcal/mol) contributed by important residues for the mutant PR-CA-p2 complex.

Residue	S_{vdW}	B_{vdW}	T_{vdW}	S_{ele}	B_{ele}	T_{ele}	S_{GB}	B_{GB}	T_{GB}	S_{SUR}	B_{SUR}	T_{SUR}	T_{GBTOT}
Leu23	-0.46	-0.05	-0.52	-1.02	0.73	-0.30	1.10	-0.75	0.35	-0.06	0.00	-0.06	-0.52
Asp25	-0.81	0.00	-0.81	-0.17	0.00	-0.17	0.37	0.00	0.37	-0.06	0.00	-0.06	-0.67
Gly27	0.00	-0.82	-0.82	0.00	-0.93	-0.93	0.00	1.21	1.21	0.00	-0.10	-0.10	-0.64
Ala28	-0.52	-0.66	-1.18	0.07	-1.66	-1.59	-0.05	0.89	0.85	-0.11	-0.06	-0.17	-2.09
Asp29	-0.60	-0.34	-0.94	8.04	0.19	8.24	-7.11	-0.65	-7.77	-0.12	-0.04	-0.16	-0.62
Ile47	-0.84	-0.34	-1.18	-0.71	1.16	0.46	0.95	-0.80	0.15	-0.15	0.00	-0.15	-0.73
Val48	-0.62	-1.29	-1.92	-0.75	-2.79	-3.53	1.04	2.13	3.16	-0.14	-0.20	-0.33	-2.62
Gly49	0.00	-1.43	-1.43	0.00	-3.03	-3.03	0.00	3.79	3.79	0.00	-0.12	-0.12	-0.80
Ile50	-2.12	-1.02	-3.14	-1.78	-0.20	-1.98	2.10	0.33	2.43	-0.25	-0.05	-0.30	-3.00
Pro81	-0.71	-0.33	-1.04	-3.58	3.08	-0.50	3.75	-2.96	0.79	-0.20	0.00	-0.20	-0.95
Ser82	-0.44	-0.40	-0.84	-1.00	1.13	0.13	1.54	-0.89	0.65	-0.12	-0.00	-0.12	-0.17
Ile84	-1.23	-0.12	-1.34	-1.11	0.93	-0.19	1.14	-0.94	0.20	-0.20	0.00	-0.20	-1.52
Arg8’	-0.09	-0.02	-0.11	-19.30	0.08	-19.21	15.16	-0.01	15.16	-0.33	0.00	-0.33	-4.49
Asp25’	-0.75	-0.10	-0.85	10.18	1.90	12.09	-8.69	-1.84	-10.53	-0.10	0.00	-0.10	0.61
Ala28’	-0.43	-0.67	-1.10	0.08	-2.09	-2.01	-0.11	2.14	2.03	-0.15	-0.05	-0.20	-1.28
Lys45’	-0.25	-0.05	-0.29	-23.24	-0.33	-23.57	24.1	0.43	24.57	-0.10	0.00	-0.10	0.61
Ile47’	-1.11	-1.12	-2.23	-2.59	-0.77	-3.35	2.55	0.24	2.80	-0.16	-0.04	-0.20	-3.00
Val48’	-1.40	-1.38	-2.77	-2.63	-0.29	-2.91	2.93	-1.53	1.40	-0.30	-0.18	-0.48	-4.77
Gly49’	0.00	-0.99	-0.99	0.00	-1.47	-1.47	0.00	1.39	1.39	0.00	-0.07	-0.07	-1.13
Ile50’	-1.19	-0.51	-1.70	-1.65	0.75	-0.91	1.67	-0.40	1.27	-0.16	-0.03	-0.19	-1.52
Phe53’	-0.23	-0.06	-0.29	-0.28	-0.50	-0.78	0.34	0.73	1.07	-0.04	0.00	-0.04	-0.04
Thr80’	-0.22	-0.12	-0.34	-0.54	0.26	-0.29	0.25	-0.14	0.12	-0.00	0.00	-0.00	-0.51
Pro81’	-0.94	-0.69	-1.63	-1.83	1.10	-0.73	2.15	-0.75	1.41	-0.18	-0.05	-0.23	-1.18
Ser82’	-0.27	-0.60	-0.86	2.32	0.76	-1.57	2.81	-0.48	2.33	-0.14	-0.01	-0.15	-0.25
Ile84’	-0.91	-0.09	-1.00	-1.02	1.15	0.14	1.09	-1.16	-0.07	-0.13	0.00	-0.13	-1.06

Table S5. Hydrogen bonds formed by two ligands DRV and CA-p2 with the WT and mutated HIV-1 PR.

		Occupancy (%)			
Donor	Acceptor	DRV-WT	DRV-mutant	CA-WT	CA-mutant
Asp25'@OD2	DRV@O18/H	100.00	100.00		
Asp30@O	DRV@N1/H	94.70	96.54		
Asp25'@OD2	DRV@O18/H	87.37	21.80		
Asp25'@OD2	DRV@N20/H		80.86		
DRV@N1	Asp30'@N/H	79.23			
DRV@O26	Asp29'@N/H	77.39			
DRV@O26	Asp30'@N/H	73.93			
DRV@O28	Asp29'@N/H	61.10			
wat@O/H	DRV@N20/H	42.16			
DRV@O10	Gly49@N/H	38.49			
Asp25'@O4	DRV@N20/H	32.99			
DRV@N1	Asp29@N/H	31.98			
wat@O	DRV@N1/H	13.85			
Gly27'@O	DRV@N20/H	10.39			
wat@O	CA-p2@N3/H			99.80	75.56
48@O	CA-p2@N1/H			98.08	99.20
27@O	CA-p2@N2/H			83.50	94.30
CA-p2@O	Asp29@N/H			78.63	43.61
48'@O	CA-p2@N5/H			62.12	97.66
CA-p2@NH2	Arg8'@NH1/H			55.19	72.10
Asp25'@OD1	CA-p2@N4/H			40.98	
Gly48'@O	CA-p2@N7/H			30.35	17.11
CA-p2@NE	Arg8'@NH2/H			29.12	87.98
Gly49'@O	CA-p2@N7/H			14.62	
Gly27'@O	CA-p2@N4/H			13.65	0.68
Ser82'@OG	CA-p2@NH2/H				73.32
46@O	CA-p2@N7/H				12.80
Pro81'@O	CA-p2@NH2/H				38.09

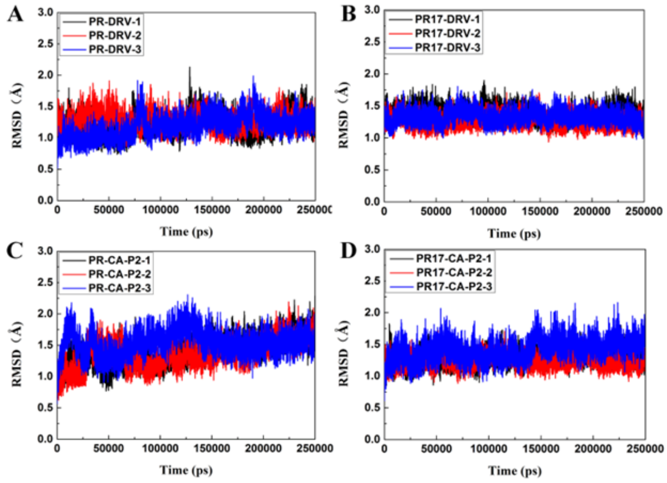

Fig. S1. Time evolution of the RMSD values relative to the initial structure in ligand bound WT PR complexes and ligand bound mutant PR complexes.

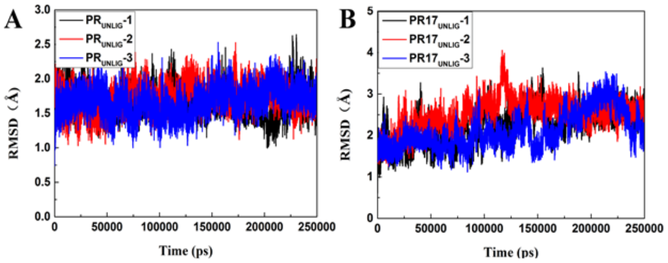

Fig. S2. Time evolution of the RMSD values relative to the initial structure of unligand PR.

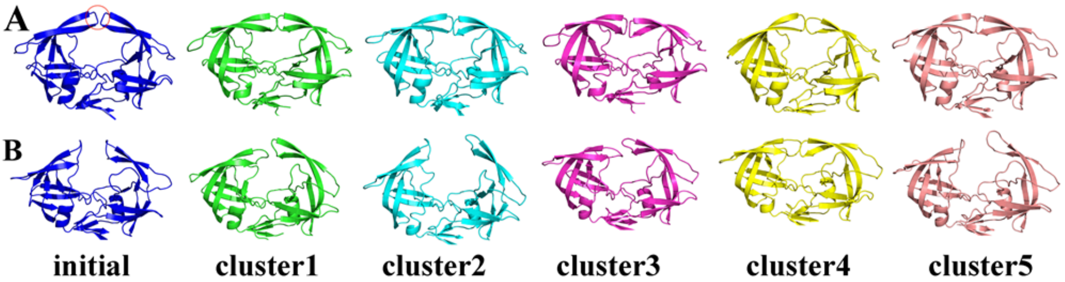

Figure S3. The initial conformation and the representative conformation extracted by clustering analysis for the unligand PR from the MD simulations: (A) unligand WT PR; (B) unligand PR^{S17}.

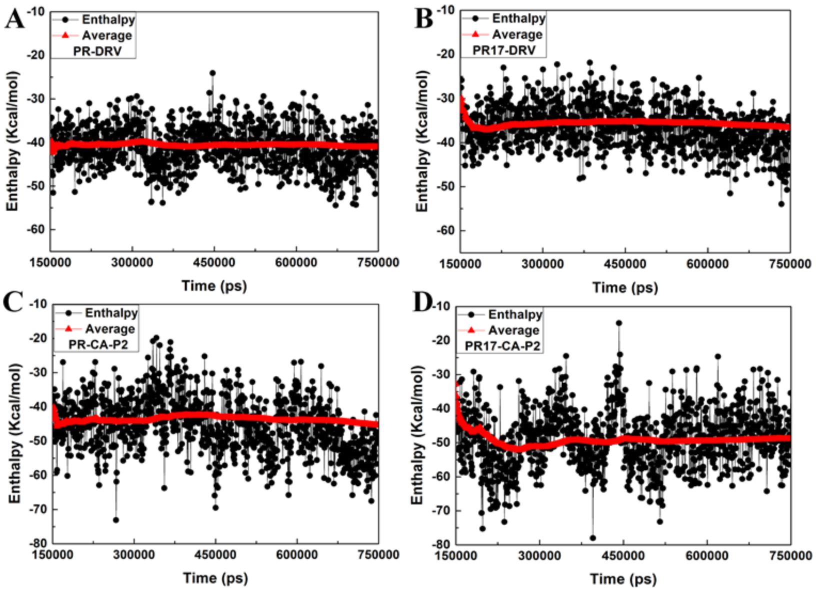

Fig. S4. The fluctuations and the accumulated mean values of enthalpies for the ligand bound WT PR complexes and ligand bound mutant PR complexes.

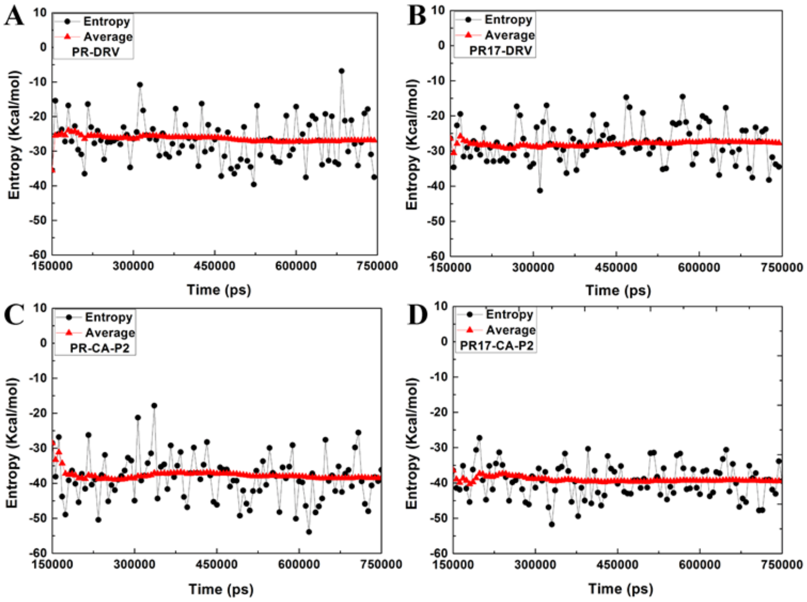

Fig. S5. The fluctuations and the accumulated mean values of entropies for the ligand bound WT PR complexes and ligand bound mutant PR complexes.

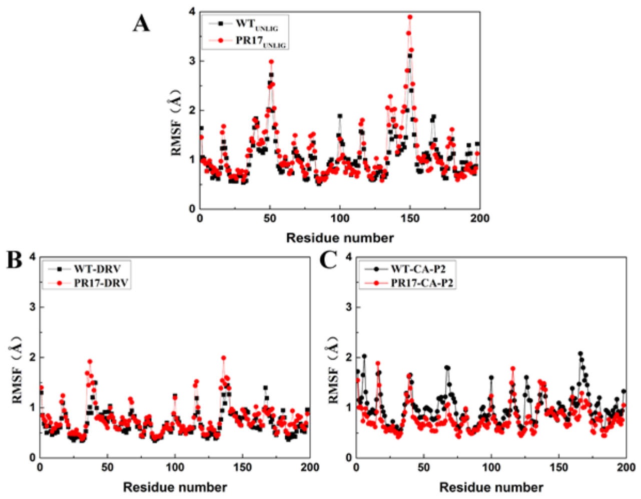

Fig. S6. RMSF of the Cα atoms versus residue number of the HIV-1 PR in the research systems.

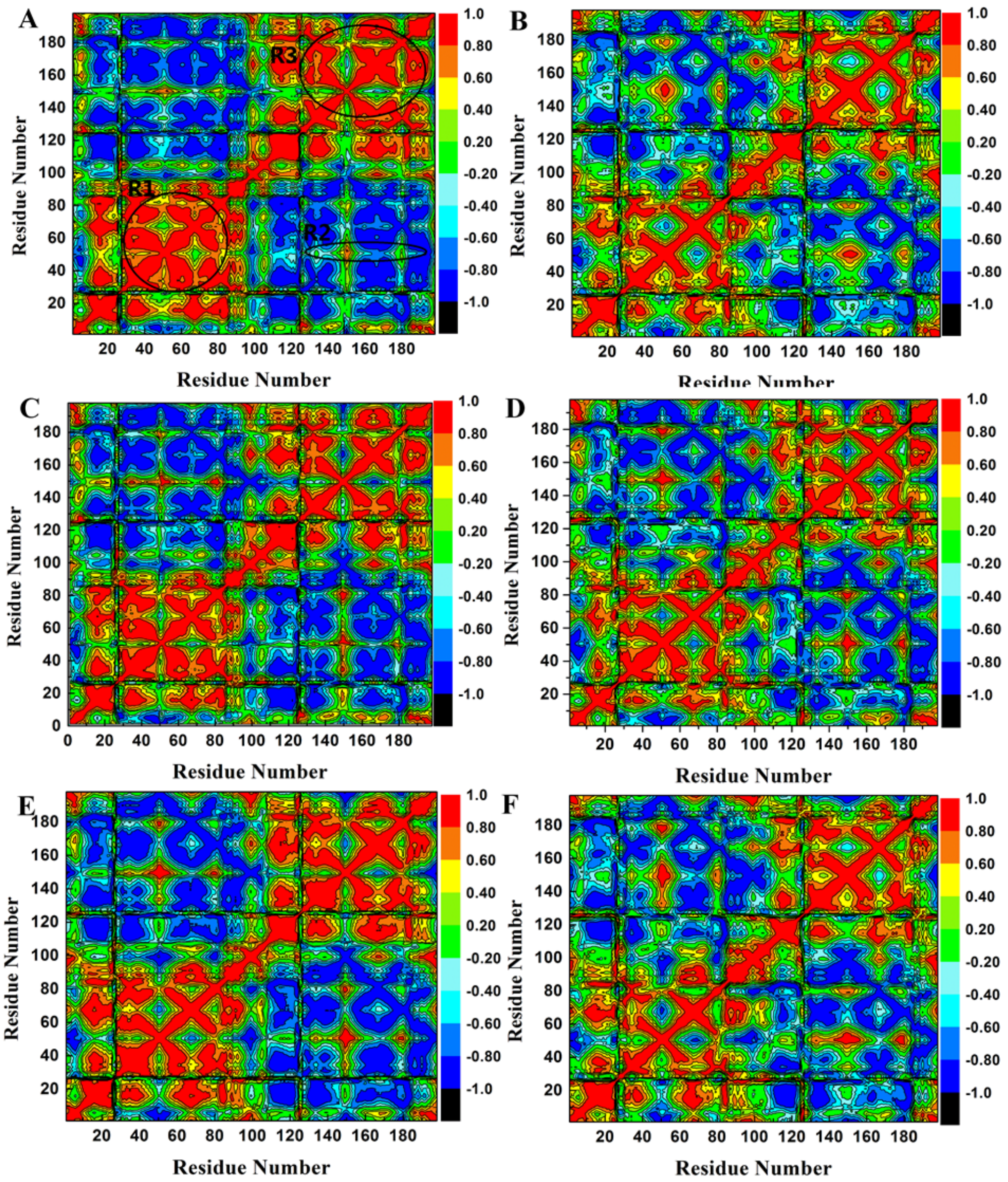

Fig. S7. Cross-correlation matrices of the fluctuations of the coordinates for Cα atoms near their corresponding average positions of the 600 ns MD simulations: (A) unligand WT PR; (B) unligand PR^{S17}; (C) DRV-WT PR; (D) DRV-PR^{S17}; (E)CA-p2-WT PR; (F) CA-p2-PR^{S17}.

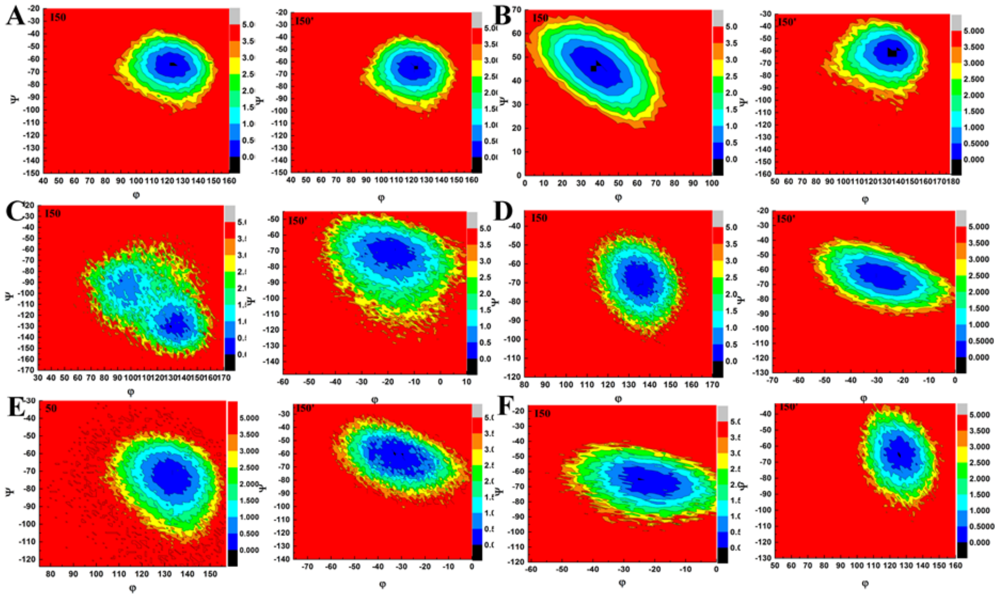

Fig. S8. Contour maps of the free energy as a function of the backbone angle φ and ψ. I50 and I50' describe the residues of the chain A and B in the PR, respectively. (A) unligand WT PR; (B) unligand PR^{S17}; (C) DRV-WT PR; (D) DRV-PR^{S17}; (E) CA-p2-WT PR; (F) CA-p2-PR^{S17}.

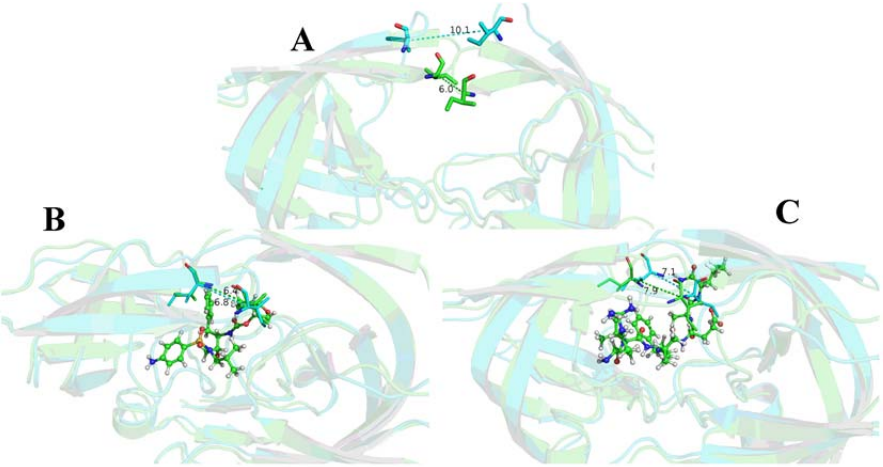

Fig. S9. Superimposed the structure of clustering analysis for the WT PR and variant PR^{S17}. The WT PR and variant PR^{S17} are shown in green and cyan, respectively. A the PR-DRV complex; B. The PR-CA-p2 complex.

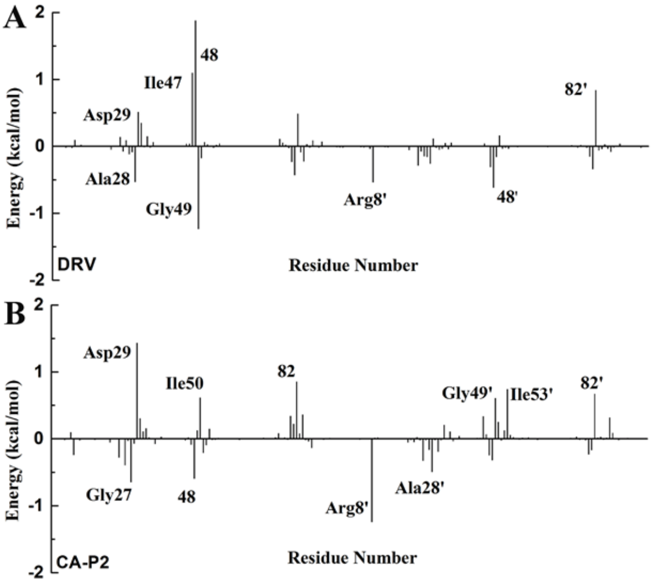

Fig. S10. The difference between the interaction enthalpy of ligands DRV and CA-p2 with the mutated and WT PR.

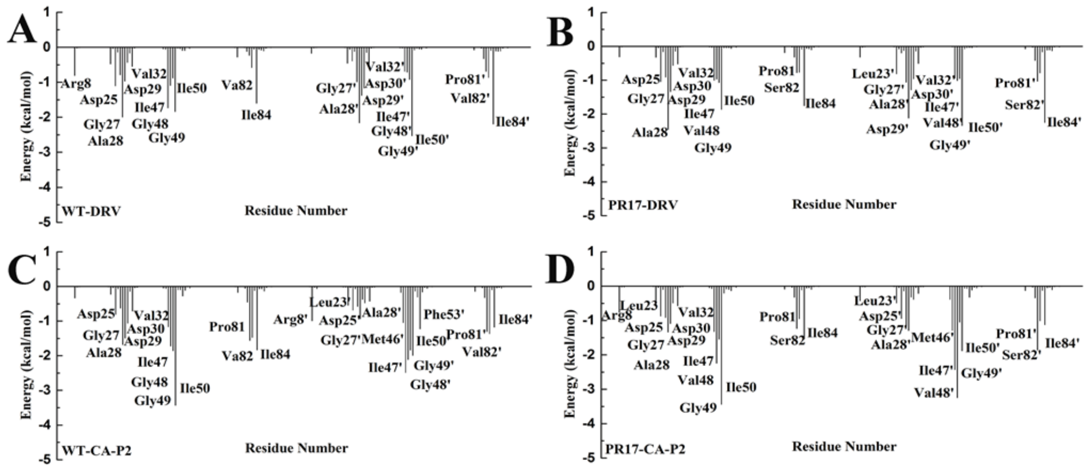

Fig. S11. The nopolar interaction energy of per-residue basis for the mutated and WT PR with ligands complexes.

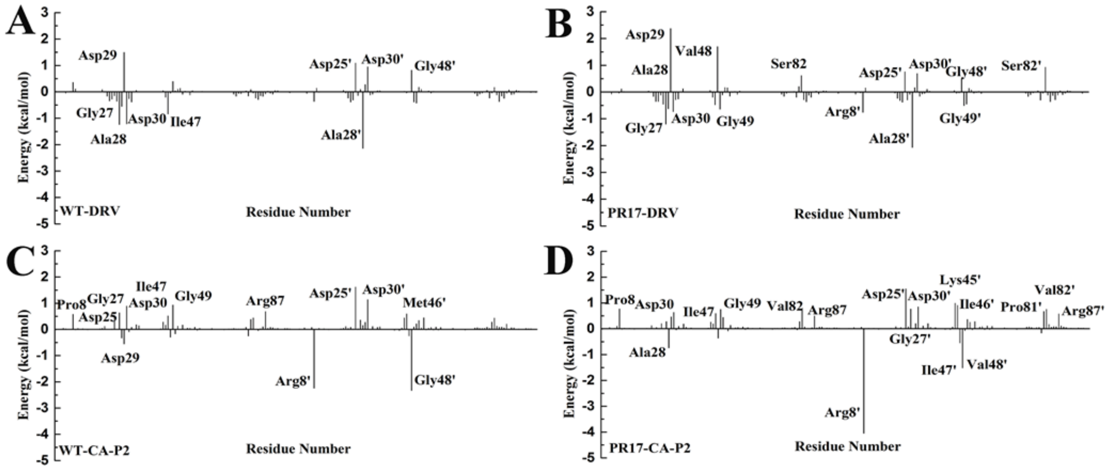

Fig. S12. The polar interaction energy of per-residue basis for the mutated and WT PR with ligands complexes.

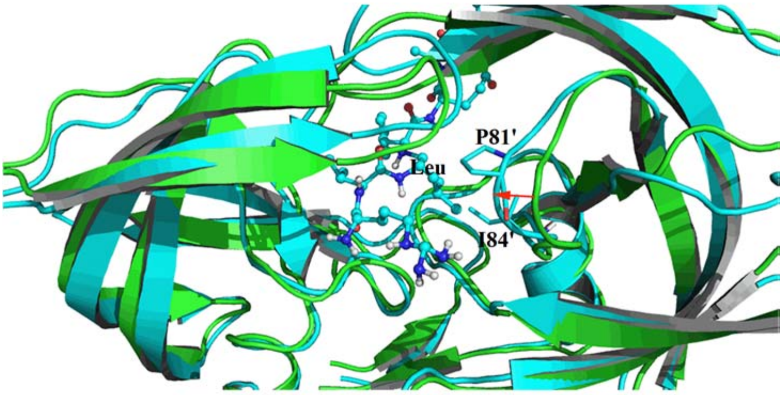

Fig. S13. Superimposed the structure of clustering analysis for the WT PR and variant PR^{S17} complexed with CA-p2. The WT PR and variant PR^{S17} are shown in green and cyan, respectively.
